# Dynamin2 functions as an accessory protein to reduce the rate of caveola internalization

**DOI:** 10.1083/jcb.202205122

**Published:** 2023-02-02

**Authors:** Elin Larsson, Björn Morén, Kerrie-Ann McMahon, Robert G. Parton, Richard Lundmark

**Affiliations:** 1 Integrative Medical Biology, Umeå University, Umeå, Sweden; 2 Umeå Centre for Microbial Research, Umeå University, Umeå, Sweden; 3 Institute for Molecular Bioscience, The University of Queensland, Brisbane, Queensland, Australia; 4 Centre for Microscopy and Microanalysis, The University of Queensland, Brisbane, Queensland, Australia; 5 Molecular Infection Medicine Sweden, Umeå University, Umeå, Sweden

## Abstract

Caveolae are small membrane invaginations that generally are stably attached to the plasma membrane. Their release is believed to depend on the GTPase dynamin 2 (Dyn2), in analogy with its role in fission of clathrin-coated vesicles. The mechanistic understanding of caveola fission is, however, sparse. Here, we used microscopy-based tracking of individual caveolae in living cells to determine the role of Dyn2 in caveola dynamics. We report that Dyn2 stably associated with the bulb of a subset of caveolae, but was not required for formation or fission of caveolae. Dyn2-positive caveolae displayed longer plasma membrane duration times, whereas depletion of Dyn2 resulted in shorter duration times and increased caveola fission. The stabilizing role of Dyn2 was independent of its GTPase activity and the caveola stabilizing protein EHD2. Thus, we propose that, in contrast to the current view, Dyn2 is not a core component of the caveolae machinery, but rather functions as an accessory protein that restrains caveola internalization.

## Teaser

Larsson et al. demonstrate that the large GTPase dynamin 2 does not mediate fission of caveolae from the plasma membrane as previously thought. Instead, dynamin 2 associates with a subset of caveolae and functions to restrain caveolae to the plasma membrane via a GTPase-independent mechanism.

## Introduction

Membrane fission is essential for the release of membrane transport vesicles in the cell. Classically, vesicles associated with the membrane via a membrane pore or neck have been seen as transient states in the process of vesicle release or fusion. However, several important processes rely on stabilization of the attached vesicle for an extended period of time (Neufeldt et al., 2018; Parton, 2018). Currently, it is largely unknown how this stabilization and temporal restrain of fission is controlled in living cells. Caveolae are small bulb-shaped invaginations of the plasma membrane (PM) with atypical lipid composition and dynamics as compared with classical membrane vesicles such as clathrin- or coat protein–coated vesicles (Parton, 2018). Caveolae are characteristically constrained to the cell surface as fully invaginated buds, but can also flatten out (Sinha et al., 2011) or undergo short-range cycles of fission and fusion (Pelkmans and Zerial, 2005).

In non-muscle cells, caveola formation is dependent on lipid-driven assembly of the integral membrane protein caveolin1 (Cav1; Monier et al., 1995; Murata et al., 1995; Rothberg et al., 1992) and the peripherally membrane attached protein, cavin1 (Aboulaich et al., 2004). Cav1 forms complexes with cholesterol in the membrane to which cavin1 is recruited, leading to invagination of such cholesterol-rich domains (Hill et al., 2008). The large ATPase EHD2, belonging to the dynamin superfamily of proteins, is present on most caveolae (Hansen et al., 2011; Morén et al., 2012; Stoeber et al., 2012). EHD2 self-oligomerizes at the caveolae neck, thereby preventing fission and constraining caveolae to the PM. Caveola stabilization is dependent on ATP binding and hydrolysis by EHD2 resulting in conformational changes that facilitate oligomerization into rings surrounding membrane tubes (Hoernke et al., 2017). Other proteins have also been shown to influence caveola dynamics. The Bin-Amphiphysin-Rvs domain containing protein pacsin2 (Pac2) binds to EHD2 and appears to aid in the stabilization of caveolae (Morén et al., 2012; Senju et al., 2015). Additionally, caveolae are tightly coupled to actin filaments, which could restrict both lateral movement and internalization (Echarri and Del Pozo, 2015). Yet, it is clear that caveolae also detach from the PM in most cell types. Different models for caveola fission have been proposed including both lipid- and protein-driven mechanisms. Accumulation of cholesterol and glycosphingolipids were shown to drive caveola fission in a process that could be counteracted by EHD2 (Hubert et al., 2020b). In addition, incorporation of cavin3 in the caveola coat was shown to increase fission (Mohan et al., 2015), and coupling of caveolae to actin via filamin was shown to promote internalization (Stahlhut and van Deurs, 2000; Sverdlov et al., 2009). In analogy with its role in clathrin-mediated endocytosis, dynamin 2 (Dyn2) has been proposed to perform fission of caveolae (Henley et al., 1998; Oh et al., 1998; Schnitzer et al., 1996).

Dyn2 is ubiquitously expressed and belongs to the dynamin superfamily of large GTPases involved in membrane fission and fusion processes. The homologs, dynamin 1 (Dyn1) and 3, are primarily expressed in the brain. Dynamins are composed of a G-domain that binds and hydrolyses GTP, the bundle signaling element, a helical stalk domain, a pleckstrin homology domain, and a proline-rich region that binds to SH3-domain containing proteins. Dynamins are known to form dimers and tetramers and to self-assemble into higher-order structures such as rings and helices. The formation of helices around membrane tubes has been extensively studied in relation to the typical role of dynamins in catalyzing fission of clathrin-coated vesicles (CCVs) for endocytosis. In the GTP-bound state, the stalk and pleckstrin homology domain bind to membranes, which facilitate self-oligomerization of dynamins into short helical rings. Nucleotide-dependent conformational changes power constriction of the helical polymer and the underlying membrane, resulting in fission. GTP hydrolysis also mediates disassembly of the helices and membrane release of dynamins (Antonny et al., 2016).

It has become apparent that dynamins can also function via other mechanisms. For example, dynamin was also identified to play a key role in the early stages of CCV formation (Aguet et al., 2013; Reis et al., 2015) where oligomerization and assembly-stimulated GTPase activity are not required (Sever et al., 2000; Sever et al., 1999). Furthermore, dynamin influences a wide range of actin-driven processes (Sever et al., 2013) and binds directly to actin (Gu et al., 2010; Palmer et al., 2015) in a mechanism independent of oligomerization and GTP binding and hydrolysis. Furthermore, the dynamin scaffolds used for membrane constriction during fission could also stabilize membrane tubules and prevent fission (Bashkirov et al., 2008; Boucrot et al., 2012; Pucadyil and Schmid, 2008). Indeed, while Dyn1 can perform scission of vesicles from planar lipid membranes, Dyn2 acts in synergy with other proteins to perform fission (Liu et al., 2011; Neumann and Schmid, 2013). In spite of the vast literature on the role of dynamin in membrane fission processes, little is known about its role at caveolae. Dyn2 is the dynamin homolog predominantly expressed in cells with caveolae. Cell-free assays of caveola budding based on purified membranes and cytosol suggested that GTP hydrolysis and dynamin were required for caveola fission (Oh et al., 1998; Schnitzer et al., 1996). Inhibition of cholera toxin B-subunit (CTxB) uptake by injection of antibodies against Dyn2 or expression of the GTPase deficient mutant K44A has been interpreted as an effect on caveola fission (Henley et al., 1998). However, CTxB is mainly internalized by other pathways (Kalia et al., 2006; Kirkham et al., 2005; Lundmark et al., 2008; Massol et al., 2004; Torgersen et al., 2001), and the regulatory role of Cav1 on CTxB uptake is independent of caveolae (Lajoie et al., 2009). The current lack of specific exogenous ligands or labels that would distinguish between surface-associated and internalized caveolae in live cells hamper studies of caveolae fission in real-time. Thus, many questions remain regarding the role of Dyn2 at caveolae and the fission of these atypical membrane invaginations.

Here, we have addressed the role of Dyn2 in caveola fission in living cells. Using a cell system with inducible expression of fluorescently labeled caveolae proteins, we show that Dyn2 is stably associated with a distinct subset of caveolae. By total internal reflection fluorescence (TIRF) microscopy and particle tracking, we have determined how caveola dynamics are influenced by Dyn2. We show that Dyn2 reduces caveola fission and increases the time that they are associated with the PM. Increased levels of Dyn2 counteract lipid-induced fission and this stabilizing effect is independent of its GTPase activity. Dyn2 acts independently of the caveola stabilizing protein EHD2 providing an additive mechanism to confine caveolae to the PM.

## Results

### Dyn2 depletion makes caveolae more dynamic in HeLa cells

**Figure 1.**
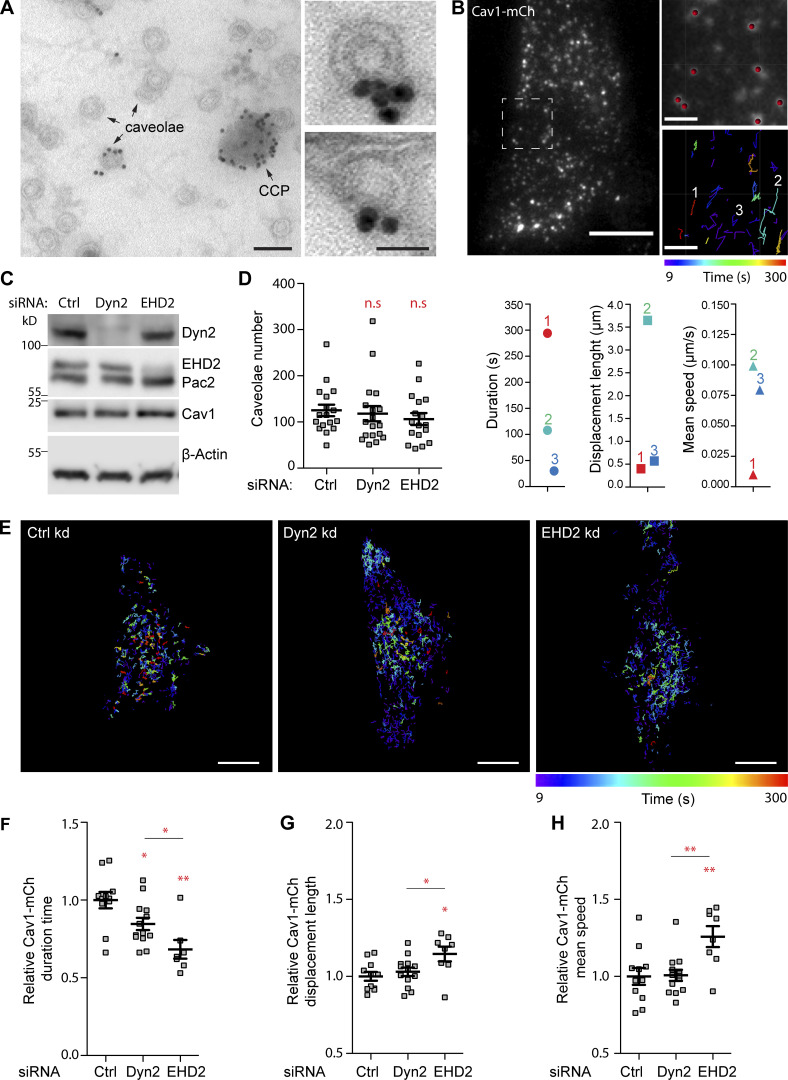
**Dyn2 depletion leads to decreased PM duration and increased mobility of caveolae in Cav1-mCh FlpIn cells. (A)** Immunogold staining of Dyn2 on PM lawns prepared from 3T3-L1 cells. Arrows indicate caveolae and a CCP. Panels to the right show caveolae at higher magnification. Bars, 100 nm main figure, 50 nm small panels. **(B)** Representative first frame image from TIRF movie of Cav1-mCh cell. Scale bar, 10 μm. Dashed white square shows the region of the cell that was tracked over 5 min. Magnifications show last frame of the movie. Scale bar, 2 μm. Red dots illustrate mCh structures recognized by the tracking program as detailed described in the Materials and methods section. The color-coded trajectories illustrate the duration and length, whereby the speed is calculated, as well as the displacement length (shortest length between the first and last position) of tracked caveolae structures for 5 min. 1 in red represents caveolae with long duration time, short displacement length, and low speed. 2 in cyan represents caveolae with intermediate duration time, long displacement length, and high speed. 3 in blue represents caveolae with short duration time, short displacement length, and high speed. **(C–H)** Cav1-mCh cells were transfected with siRNA for 72 h before experiments were performed. **(C)** Immunoblots of cells treated with siRNAs as indicated. **(D)** Number of caveolae counted in the basal PM in cells imaged on TIRF. Mean ± SEM from at least 17 cells per condition. **(E)** Color-coded trajectories of caveolae in Cav1-mCh cells treated with siRNA as indicated. Cells were imaged by TIRF over 5 min. Scale bars, 10 μm. **(F–H) **Quantification of Cav1-mCh track duration time (F), speed (G), and displacement length (H). Numbers were related to ctrl-treated cells. Analyses were performed using Imaris software and track mean ± SEM from at least eight cells per condition are shown. Significance was assessed using *t* test, *P ≤ 0.05, **P ≤ 0.01. Source data are available for this figure: SourceData F1.

**Figure S1.**
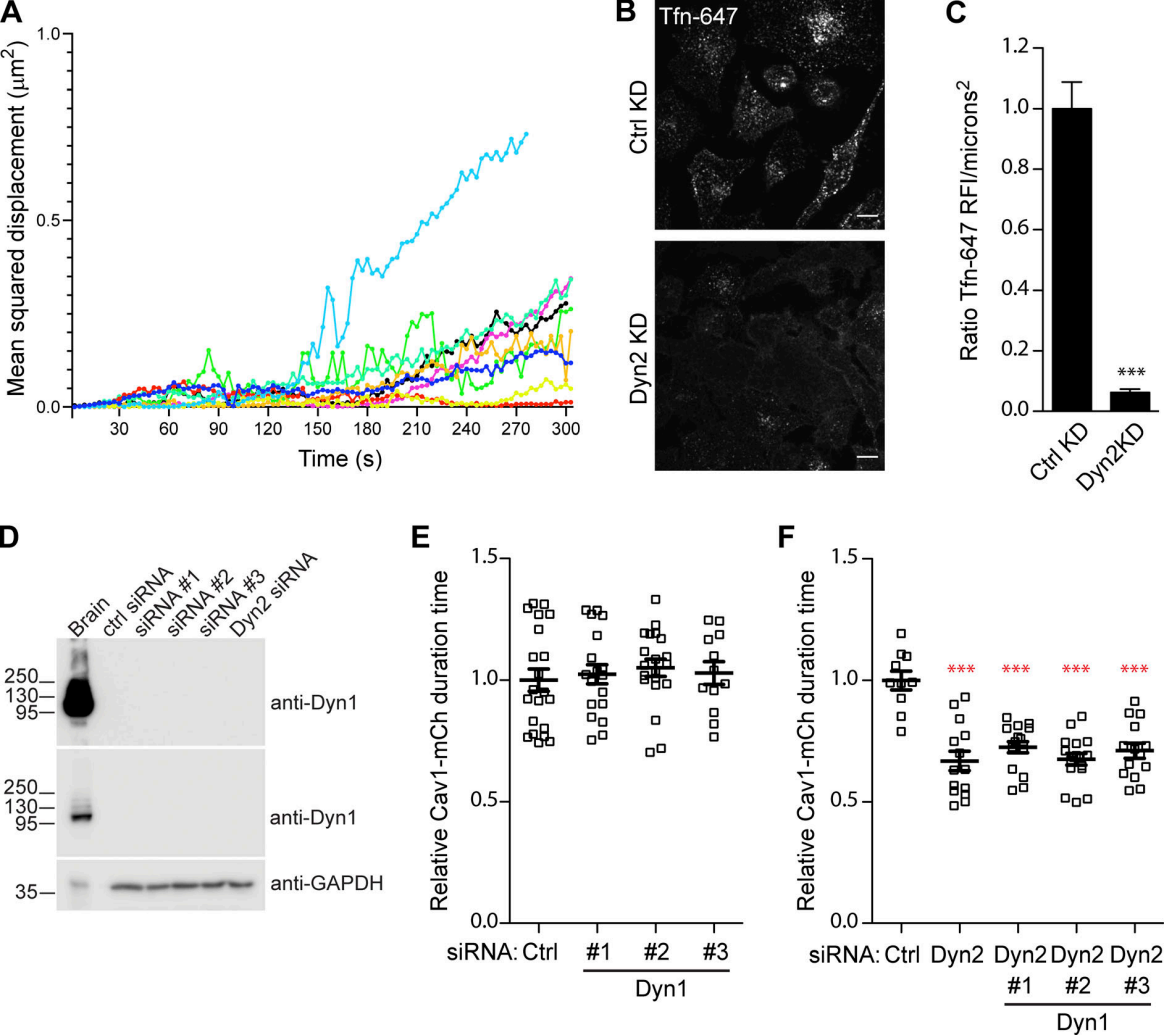
**Analysis of Tf-647 uptake and caveolae track duration time in Cav1-mCh FlpIn cells depleted of Dyn1 or Dyn2. (A)** Analysis of mean squared displacement over time of nine stable Cav1-mCh–positive caveolae. **(B)** Representative images of fixed Cav1-mCh cells transfected with siRNA as indicated for 72 h before 10 min incubation with Alexa fluor 647 conjugated Tf (Tf-647; 5 μg/ml) at 37°C. Scale bar, 10 μm. **(C)** Quantification shows relative Tf-647 fluorescent intensity/microns^2^. At least 40 cells were quantified per condition, significance was assessed using *t* test, ***P ≤ 0.001. **(D)** Immunoblots of whole cell lysate of Cav1-mCh cells treated with siRNA as indicated for 72 h prior to harvest. As a positive control protein, lysate of rat brain was loaded. Two exposures of the blot probed with rabbit anit-Dyn1 are shown to illustrate the extreme difference of Dyn1 levels present in the different cell types. Molecular weights in kD. **(E and F)** Quantification of the Cav1-mCh track duration time in cells depleted of Dyn1 using three independent siRNAs as indicated (E) or cells depleted of both Dyn1 and Dyn2 using siRNA as indicated. GAPDH served as a loading control (F). Numbers were related to ctrl siRNA treated cells. Analyses were performed using Imaris software, and track mean ± SEM from at least 12 (E) or 10 (F) cells per condition are shown. Significance was assessed using *t* test, ***P ≤ 0.001. Source data are available for this figure: SourceData FS1.

In agreement with the proposed role of Dyn2 at caveolae (Oh et al., 1998), immunogold labeling of PM lawns of differentiated 3T3-L1 cells confirmed the presence of Dyn2 at a subset of the numerous caveolae in these cells (Fig. 1 A). However, compared to the localization at clathrin-coated pits (CCPs), the Dyn2 labeling was sparse. To further investigate the proposed role of Dyn2 in caveola fission, we tracked real-time caveola dynamics using a HeLa Cav1-mCh FlpIn cell line (Hubert et al., 2020b). In these cells, expression of mCherry-tagged Cav1 (Cav1-mCh) is induced to near endogenous levels following addition of doxycycline. TIRF imaging allowed restricted visualization of caveolae at or in close proximity to the PM (Fig. 1 B). TIRF movies were analyzed using the Imaris tracking software, where Cav1-positive spots of a certain size and fluorescent intensity were regarded as caveolae (Fig. 1 B and Video 1). Tracking showed that the caveolae within a cell display a wide range of dynamics, as previously described (Video 1; Boucrot et al., 2011; Hoernke et al., 2017; Hubert et al., 2020b; Mohan et al., 2015). The combination of the dynamic parameters (i) duration time, (ii) displacement length (shortest length between the first and last position), and (iii) mean speed is characteristic of the dynamic behavior of individual caveola (Fig. 1 B). Long duration times, short displacement length, and low-track mean speed are indicative of caveolae stably connected to the cell surface (Fig. 1 B, line 1 and red symbols). Analysis of the mean squared displacement showed that such caveolae displayed a slow diffusional lateral movement with occasional leaps of directed motion (Fig. S1 A). Intermediate duration time, high speed, and long displacement length are interpreted as fissioned caveolae moving rapidly in the plane of the membrane (Fig. 1 B, line 2 and cyan symbols). Short duration times, short displacement length, and high mean speed are indicative of fissioned caveolae moving rapidly in and out of the TIRF plane (Fig. 1 B, line 3 and blue symbols). Since freely diffusing caveolae can appear in the TIRF plane several times during the imaging period, in combination with small fluctuations in the TIRF plane causing false positive or divided tracks, the number of short duration tracks is overrepresented. Therefore, we do not consider average values as absolute. Yet, by analyzing all detected caveolae in many cells without biased selection of data, we are able to compare the average dynamic parameters between different experimental conditions.

**Video 1.** **Single particle tracking of Cav1-mCh in a HeLa Cav1-mCh FlpIn cell.** Representative single particle tracking of Cav1-mCh structures of a HeLa Cav1-mCh cell treated with ctrl siRNA imaged on TIRF every third s for 5 min. The mCh fluorescence was segmented using Imaris 9.5.1, and white spheres represents caveolae positive for Cav1-mCh. Rainbow-colored trajectories display duration time and displacement length of the caveolae as they are being traced. Purple trajectories represent short duration times (9–15 s) whereas red trajectories represent caveolae with long duration times (291–300 s). Frame rate: 10 frames per second.

To determine the role of Dyn2 in regulating caveola dynamics, cells were depleted of Dyn2 using siRNA (Fig. 1 C) prior to TIRF microscopy analysis. For comparison, cells were also treated with control siRNA or siRNA against EHD2. Quantification of the number of caveolae at the basal membrane did not reveal any significant change in cells depleted of dynamin or EHD2 as compared with control (ctrl)-treated cells (Fig. 1 D). Analysis of the dynamic track parameters confirmed that EHD2 depletion, in accordance with its role in stabilizing caveolae at the PM (Morén et al., 2012; Stoeber et al., 2012), resulted in less stable and more fissioned caveolae as compared with ctrl-treated cells (Fig. 1, E–H, and Video 2). Surprisingly, dynamin knockdown resulted in a 20% overall decrease in the duration time, showing that similar to EHD2 depletion, loss of Dyn2 decreased the time that caveolae spent at the PM (Fig. 1, E–H, and Video 3). However, in contrast to EHD2-depleted cells, there was only a slight difference in the speed or displacement as compared with control cells. The uptake of transferrin (Tf) was severely impaired in Dyn2-depleted cells, consistent with CCV fission being blocked (Fig. S1, B and C). Although low levels of Dyn2 could be remaining in these cells following siRNA depletion, these data suggested that Dyn2 is not needed for caveola fission, but that it rather stabilizes the PM association of caveolae. The Dyn2 homolog Dyn1 has been reported to be activated to mediate compensatory clathrin-mediated endocytosis in non-neuronal cells (Reis et al., 2015). However, consistent with Dyn2 depletion abolishing Tf uptake, the expression of Dyn1 is very low in HeLa cells, and we were not able to detect Dyn1 in these cells by immunoblotting (Fig. S1 D). Knockdown of Dyn1 using three independent siRNAs did not significantly affect dynamic parameters of caveolae as compared with ctrl cells (Fig. S1 E). Furthermore, the effects of double knockdown of both Dyn1 and Dyn2 on caveola dynamics were identical to those observed in single Dyn2 knockdown cells, showing that Dyn1 does not play a significant role in regulating caveola dynamics in these cells (Fig. S1 F).

**Video 2.** **EHD2 depletion increases caveola dynamics.** Representative single particle tracking of Cav1-mCh structures of a HeLa Cav1-mCh cell treated with EHD2 siRNA imaged on TIRF every third s for 5 min. The mCh fluorescence was segmented using Imaris 9.5.1, and white spheres represent caveolae positive for Cav1-mCh. Rainbow-colored trajectories display duration time and displacement length of the caveolae as they are being traced. Purple trajectories represent short duration times (9–15 s) whereas red trajectories represent caveolae with long duration times (291–300 s). Frame rate: 10 frames per second.

**Video 3.** **Dyn2 depletion increases caveola dynamics.** Representative single particle tracking of Cav1-mCh structures of a HeLa Cav1-mCh cell treated with Dyn2 siRNA imaged on TIRF every third s for 5 min. The mCh fluorescence was segmented using Imaris 9.5.1, and white spheres represent caveolae positive for Cav1-mCh. Rainbow-colored trajectories display duration time and displacement length of the caveolae as they are being traced. Purple trajectories represent short duration times (9–15 s) whereas red trajectories represent caveolae with long duration times (291–300 s). Frame rate: 10 frames per second.

### Dyn2 confines caveolae to the PM

**Figure 2.**
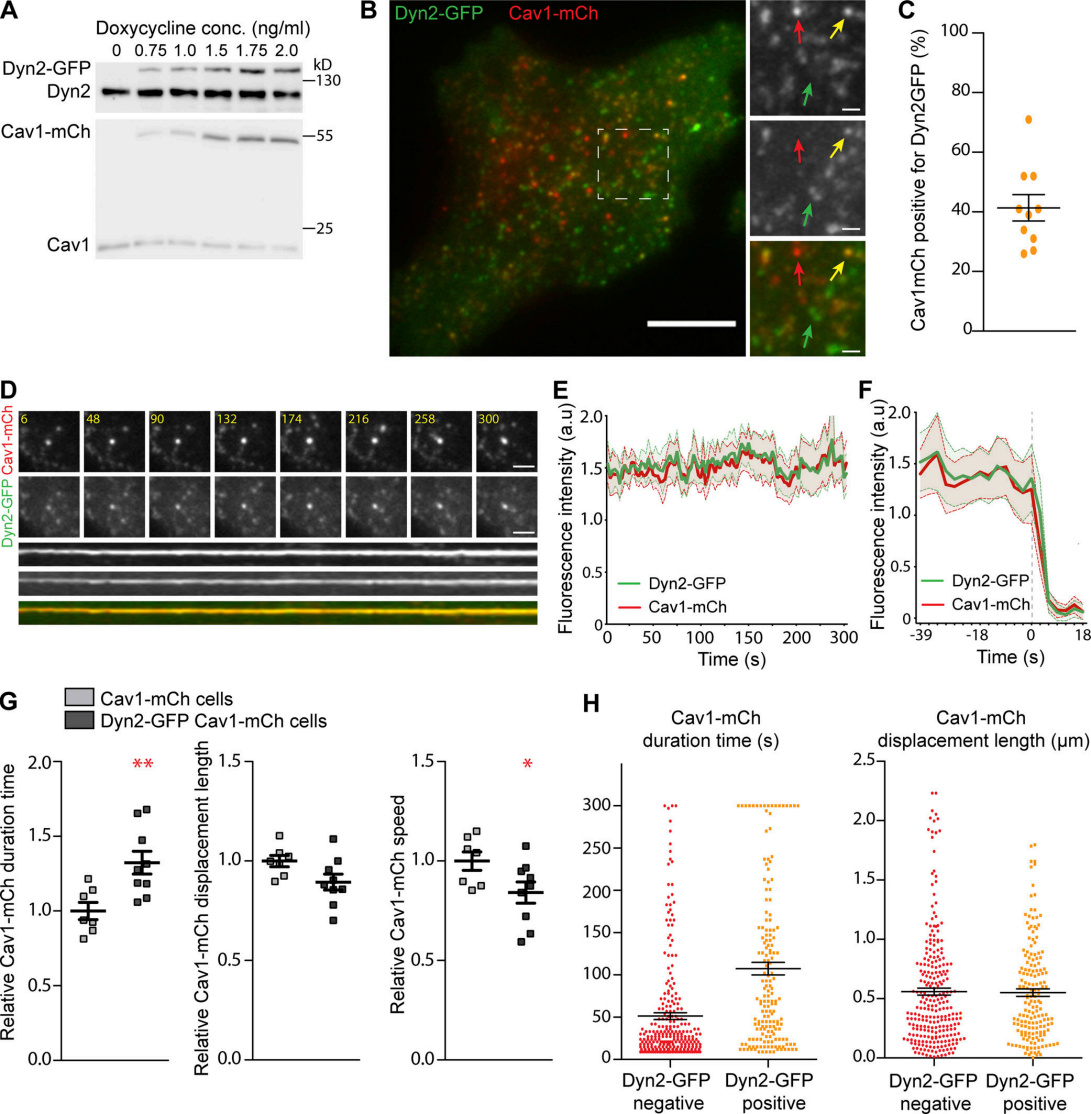
**Dyn2-GFP increases PM duration of caveolae but does not influence their mobility. (A)** Immunoblots of Dyn2-GFP-Cav1-mCh cells treated with concentrations of doxycycline as indicated. Doxycycline concentration used for further experiments was 1.0 ng/ml. **(B)** Representative image from TIRF movie of Dyn2-GFP-Cav1-mCh cell. Red arrow highlights structure only positive for Cav1-mCh, yellow arrow highlights structure positive for both Cav1-mCh and Dyn2-GFP, and green arrow highlights structures only positive for Dyn2-GFP. Scale bar, 10 and 2 μm for magnification. **(C)** Quantification of percentage of caveolae positive for Dyn2-GFP. Data from 10 cells are shown as scatter dot plot, mean ± SEM. **(D)** Top, TIRF image series of stable caveolae positive for both Cav1-mCh and Dyn2-GFP. Time in seconds as indicated. Scale bar, 2 μm. Bottom, kymograph showing stable association of Cav1-mCh and Dyn2-GFP to the plasma membrane during 300 s in TIRF movie. **(E and F)** Quantification of the fluorescence intensity in arbitrary units (a.u) of mCh and GFP of caveolae positive for both Cav1-mCh and Dyn2-GFP over time. **(E)** Fluorescence intensity from 10 stable caveolae over 300 s, mean ± SEM (shaded area). **(F)** Fluorescence intensity of fissioned caveolae, time point 0 s represent the last frame of caveolae before fission. Mean ± SEM (shaded area) from nine fissioned caveolae). **(G)** Quantification of Cav1-mCh track duration time, displacement length and mean speed in Cav1-mCh cells (gray) and Dyn2-GFP-Cav1-mCh cells (black). Analysis was performed using Imaris software, and track mean from at least 10 cells per condition is shown ± SEM. **(H)** Caveolae track duration times and displacement lengths divided in pools of Dyn2-GFP negative (red) or positive (orange). Dyn2-GFP-Cav1-mCh cells were imaged on TIRF over 5 min. Cav1-mCh spots were followed throughout the time series and scored whether they were positive or negative for Dyn2-GFP. Scatter dot plots show all Cav1-mCh tracks from three different cells. Analysis was performed using Imaris software, and data are shown as mean ± SEM. Significance was assessed using *t* test, *P ≤ 0.05, **P ≤ 0.01. Source data are available for this figure: SourceData F2.

**Figure S2.**
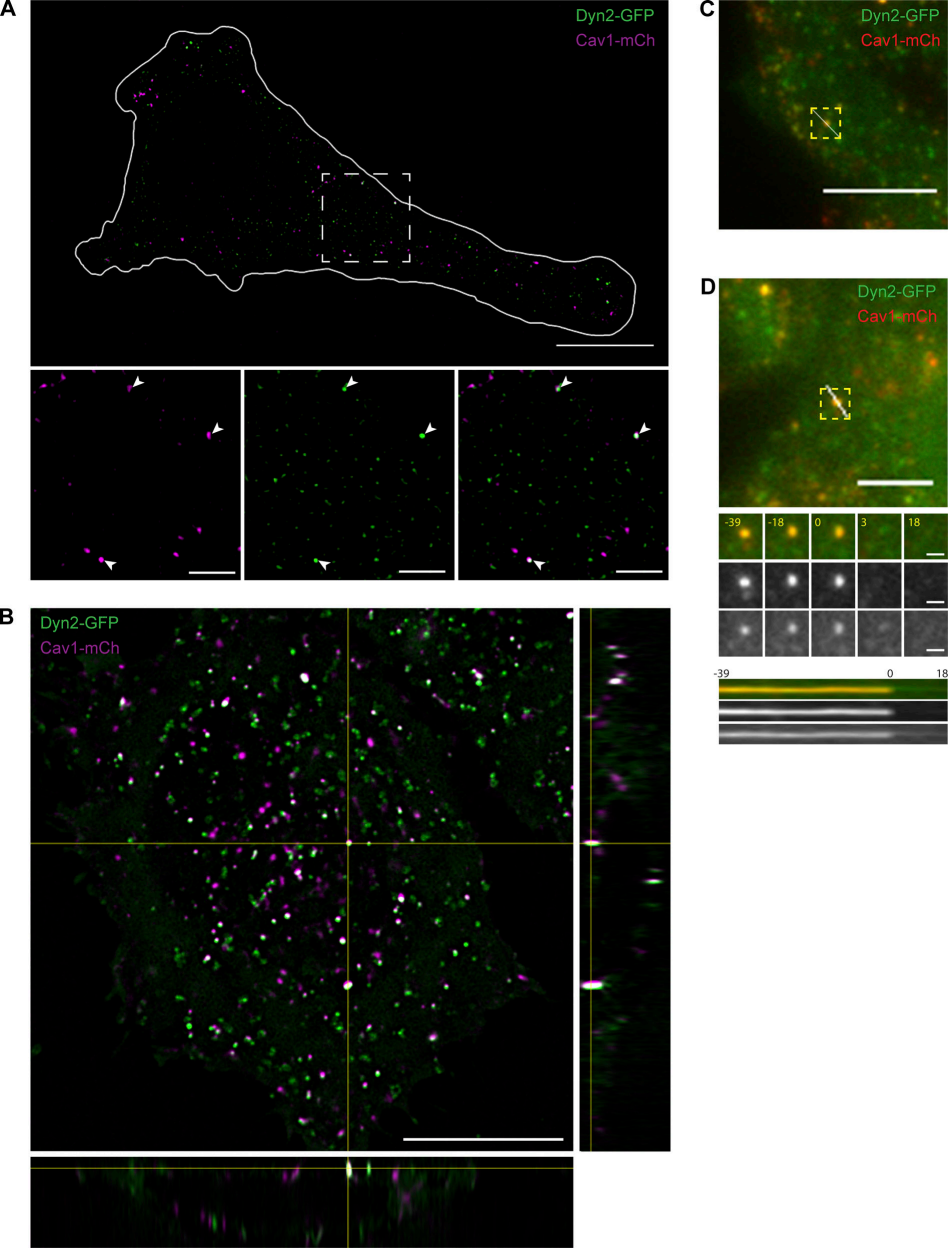
**SIM analysis of the colocalization between Dyn2-GFP and Cav1-mCh. (A)** Representative SIM2 image of Dyn2-GFP-Cav1-mCh FlpIn cell imaged live. The white line marks the outline of the cell and the dashed square marks the location of the magnified areas in the bottom panel. White arrowheads highlight structures positive for both Dyn2-GFP and Cav1-mCh. Scale bar, 10 or 2 μm for magnification. **(B)** Orthogonal view of a live Dyn2-GFP-Cav1-mCh cell imaged with Lattice SIM2. Scale bar, 10 μm. **(C)** Dyn2-GFP-Cav1-mCh cell with line that indicates location of kymograph in Fig. 2 D. Scale bar, 5 μm. **(D)** Dyn2-GFP-Cav1-mCh cell with line that indicates the location of the kymograph presented at the bottom. Scale bar, 5 or 1 μm for magnification. Insets show TIRF image series of a caveola positive for both Cav1-mCh and Dyn2-GFP that disappear from the TIRF field. Time in seconds as indicated.

To follow up on the potential stabilizing role of Dyn2 at caveolae and enable visualization in real-time, we constructed a HeLa FlpIn cell line where the expression of Dyn2-EGFP (Dyn2-GFP) and Cav1-mCh could be induced to sub or near endogenous levels (Fig. 2 A). TIRF time-lapse movies of the cells showed that the majority of the GFP-labeled Dyn2 did not colocalize with caveolae as expected due to its recruitment to clathrin-coated vesicles (Fig. 2 B). Yet, in addition to caveolae only, positive for Cav1-mCh, we detected a distinct pool of caveolae where Dyn2-GFP colocalized with Cav1-mCh (Fig. 2 B) in accordance with previous results (Fig. 1 A). In these cells, which express elevated levels of Dyn2, quantification suggested that this fraction represented ∼40% of the caveolae (Fig. 2 C). The distinct colocalization to a subset of caveolae was confirmed by super resolution structured illumination microscopy (SIM; Fig. S2, A and B, and Video 4). Dynamin has been shown to accumulate in a burst of fluorescence intensity coinciding with fission of CCVs (Merrifield et al., 2002). However, kymographic analysis of the colocalization over time at caveolae showed that Dyn2 was stably associated with caveolae (Fig. 2 D and Fig. S2 C). The fluorescent intensity profile of Dyn2-GFP and Cav1-mCh showed correlated fluctuations indicative of stable levels of Dyn2-GFP at caveolae over time (Fig. 2 E). There was also no burst in Dyn2 fluorescence coinciding with caveolae undergoing fission leaving the TIRF plane (Fig. 2 F and Fig. S2 D). These results show that the mere presence of Dyn2 at caveolae does not lead to fission, and that fission is not coupled to Dyn2 accumulation.

**Video 4.** **Localization of Dyn2-GFP to caveolae.** Dyn2-GFP-Cav1-mCh cells were induced with 1 ng/ml Dox and imaged using SIM (SIM^2^ algorithm). Cells were imaged every 60 ms for a total of 100 frames. Green channel represents Dyn2-GFP and magenta represents Cav1-mCh. Frame rate: 10 frames per second.

To study if the elevated levels of dynamin affected the time caveolae spent at the cell surface, Cav1-mCh spots were tracked and the dynamic parameters were analyzed (Fig. 2 G). We found that the track duration time in cells expressing Dyn2-GFP increased by around 30% as compared with caveolae tracks in Cav1-mCh FlpIn cells (Fig. 2 G). Furthermore, we observed a decrease in the displacement length and mean speed (Fig. 2 G), further supporting that Dyn2 stabilizes caveolae and prevents fission. As elevated global levels of Dyn2 might have an overall effect on the cell, and thereby indirectly increase the caveola stability, we aimed to determine if the stabilizing effect was specific and directly coupled to the localization of Dyn2 at caveolae. Caveolae were divided into two pools, positive or negative for Dyn2-GFP as determined by kymographic analysis, and the track duration time for each pool was measured and plotted (Fig. 2 H). Caveolae positive for Dyn2-GFP displayed a mean duration time that was more than twice as long as the pool that lacked Dyn2-GFP, suggesting that Dyn2 stabilized caveolae at the PM. Interestingly, the displacement length of the different pools was not significantly different in these cells as compared with control (Fig. 2 H). This agrees with the finding that knockdown of Dyn2 did not have a significant effect on the displacement length and suggests that Dyn2 regulates cell surface duration of caveolae without influencing the directional lateral movement.

### Dyn2 recruitment to caveolae is not mediated by EHD2 and Pac2

**Figure S3.**
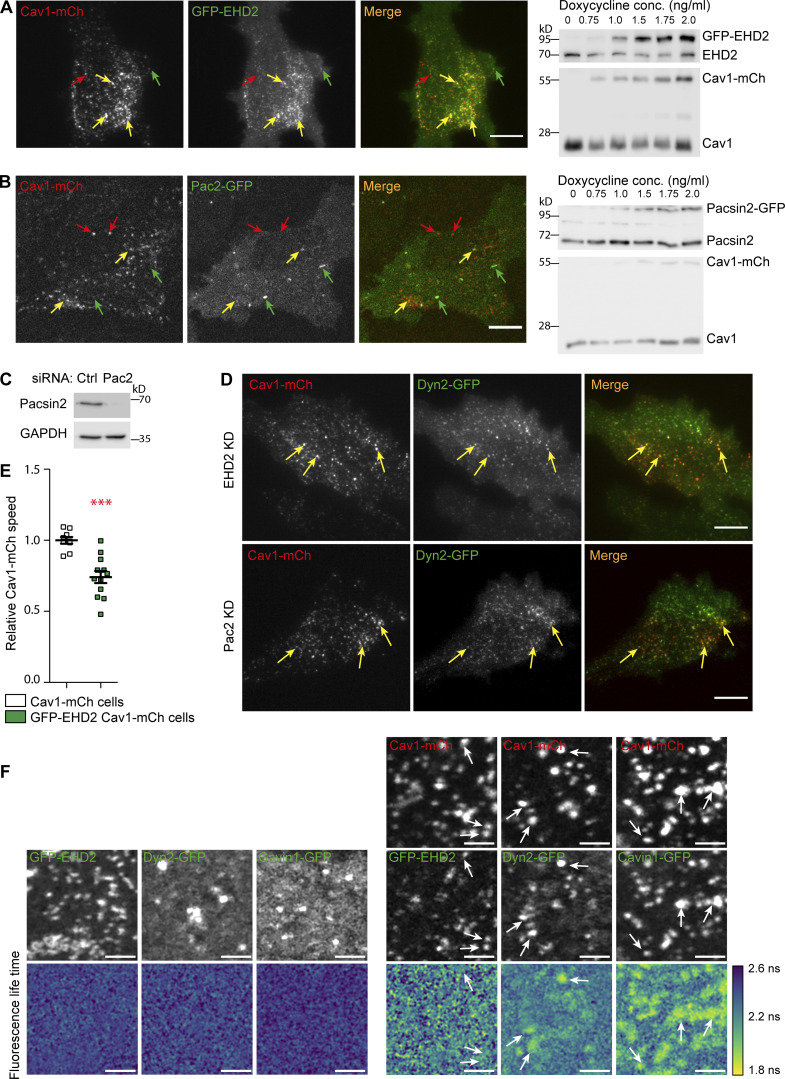
**Characterization of GFP-EHD2-Cav1-mCh and Pac2-GFP-Cav1-mCh HeLa FlpIn cells and localization of Dyn2-GFP following EHD2 or Pac2 depletion. (A)** Representative image from TIRF movie of a GFP-EHD2-Cav1-mCh cell. Red arrow depicts structures only positive for Cav1-mCh, green arrow highlights spots only positive for GFP-EHD2, and yellow arrows highlight structures positive for both GFP-EHD2 and Cav1-mCh. Immunoblot of GFP-EHD2-Cav1-mCh cells treated with concentrations of doxycycline as indicated. Doxycycline concentration used for further experiments were 1.0 ng/ml. **(B)** Representative image from TIRF movie of Pac2-GFP-Cav1-mCh cell. Red arrow depicts structures only positive for Cav1-mCh, green arrow highlights spots only positive for Pac2-GFP, and yellow arrows highlight structure positive for both Pac2-GFP and Cav1-mCh. Immunoblot of Pac2-GFP-Cav1-mCh cells treated with concentrations of doxycycline as indicated. Doxycycline concentration used for further experiments was 1.0 ng/ml. **(C)** Immunoblots of cells treated with siRNAs as indicated. GAPDH served as a loading control. **(D)** Representative image from TIRF movie of Dyn2-GFP-Cav1-mCh cells treated with siRNA directed against EHD2 or Pac2 as indicated. Yellow arrows highlight caveolae positive for Dyn2-GFP. **(E)** Quantification of Cav1-mCh track mean speed in GFP-EHD2-Cav1-mCh cells (green). Numbers were related to Cav1-mCh cells (white). Track mean ± SEM from at least seven cells per condition are shown. Significance was assessed using *t* test, ***P ≤ 0.001. All scale bars, 10 μm. **(F)** FLIM-FRET of GFP-fusion proteins. Top images show fluorescence micrographs of cells expressing GFP-fusion proteins and Cav1-mCh as indicated. Bottom of the two panels show EGFP fluorescence lifetime. White arrows highlight structures where EGFP and mCherry colocalize. Scale bar, 2 μm. Source data are available for this figure: SourceData FS3.

**Figure 3.**
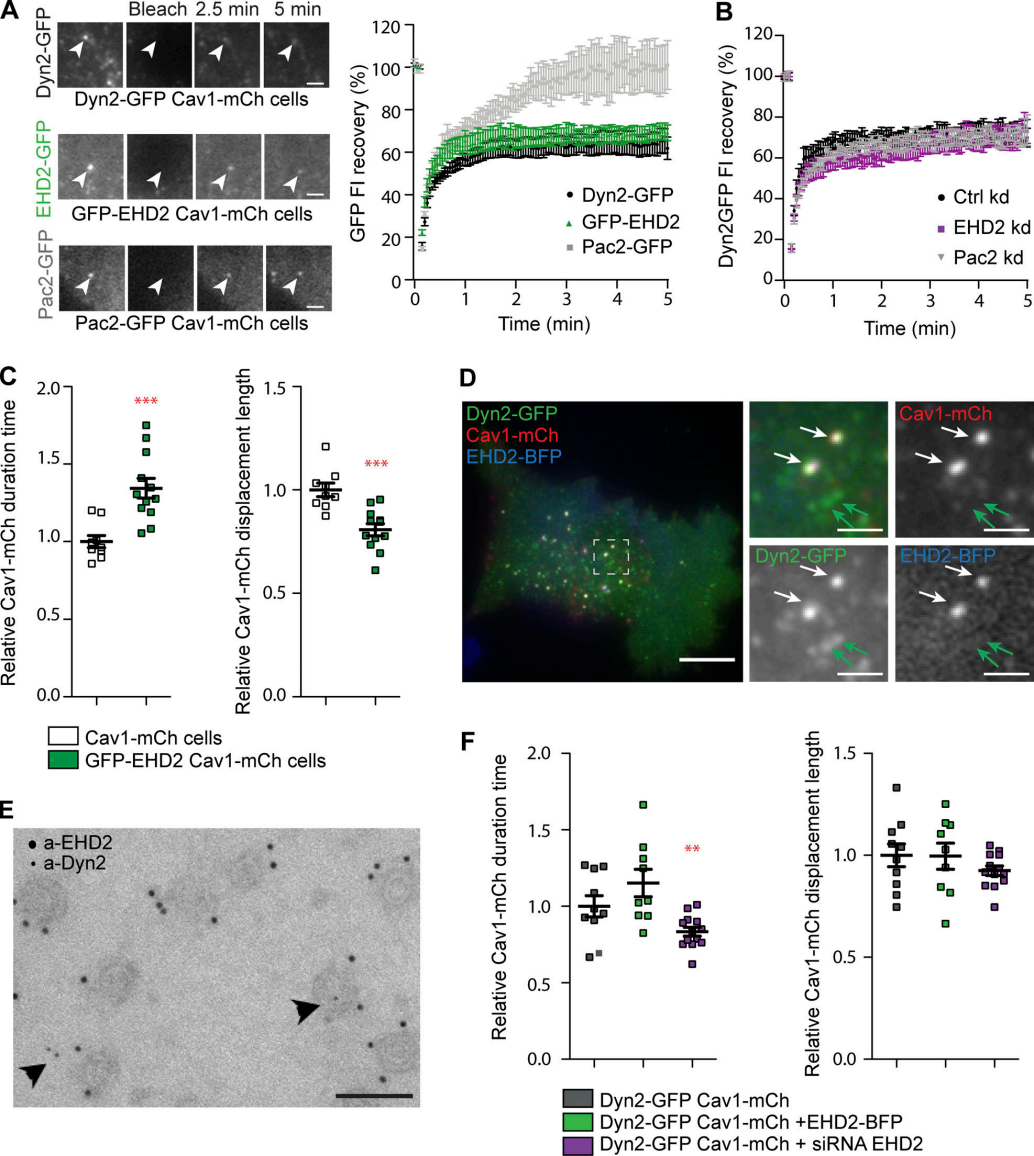
**Dyn2 and EHD2 stabilize caveolae via different but cooperative mechanisms. (A)** Image panel showing GFP recovery after photobleaching in Dyn2GFP-Cav1-mCh cells, GFP-EHD2-Cav1-mCh cells, or Pac2-GFP-Cav1-mCh cells. Graph shows percentage GFP-intensity recovery curves after photobleaching of mCh and GFP colocalizing structures at the PM. Arrowheads depict caveolae prior to and after photobleaching as indicated in the figure. Scale bar, 2 μm. **(B)** Recovery curves of Dyn2-GFP intensities after photobleaching of caveolae in Dyn2-GFP-Cav1-mCh cells treated with Ctrl, EHD2, or Pac2 siRNA as indicated. **(C)** Quantification of Cav1-mCh track duration time and displacement length in GFP-EHD2-Cav1-mCh cells (green). Numbers were related to Cav1-mCh cells (white). Track mean ± SEM from at least seven cells per condition are shown. **(D)** Representative image from TIRF movie of Dyn2-GFP-Cav1-mCh cells transiently expressing EHD2-BFP. White arrows highlight structures where all three tagged proteins are colocalized, and green arrows highlights structures only positive for Dyn2-GFP. Scale bar, 10 or 2 μm for magnification. **(E)** Immunogold labeling of EHD2 (large gold) and Dyn2 (small gold) on PM lawns prepared from 3T3-L1 cells. Arrowheads depict caveolae positive for both EHD2 and Dyn2. Scale bar, 100 nm. **(F)** Quantification of Cav1-mCh track duration time and displacement length in Dyn2-GFP-Cav1-mCh cells (black), transiently expressing EHD2-BFP (dark green), or depleted from EHD2 (purple). Numbers were related to Dyn2-GFP-Cav1-mCh cells (black). Track mean ± SEM from at least nine cells per condition are shown. Significance was assessed using *t* test, **P ≤ 0.01, ***P ≤ 0.001.

Like Dyn2, EHD2 and Pac2 have been shown to influence caveola dynamics (Hubert et al., 2020a). To be able to further compare the role of these proteins at caveolae, we constructed GFP-EHD2-Cav1-mCh and Pac2-Cav1-mCh HeLa FlpIn cell lines (Fig. S3, A and B). GFP-EHD2 was detected on most (84 ± 2.5%) caveolae in agreement with previous results (Fig. S3 A; Morén et al., 2012; Stoeber et al., 2012). Pac2-GFP was observed to tubulate membrane devoid of Cav1-mCh, but also to colocalize to Cav1-mCh, however not to the same extent as Dyn2 or EHD2 (Fig. S3 B). To compare the turnover rates at caveolae, Dyn2-GFP, GFP-EHD2, or Pac2-GFP were photobleached at caveolae in the respective cell lines, and the fluorescence recovery was recorded and plotted (Fig. 3 A). All three proteins showed a similar initial rate of recovery following photobleaching. However, 5 min after photobleaching, only ∼60% of the fluorescent signal had recovered for both Dyn2 and EHD2 (Fig. 3 A). The low recovery showed that there is a large, stably associated pool of both Dyn2 and EHD2 at caveolae. In comparison, the entire pool of fluorescent Pac2 had been exchanged after 5 min (Fig. 3 A). This shows that Dyn2 and EHD2 are firmly associated with caveolae.

Dynamin has been suggested to interact with EHDs and pacsins (Jakobsson et al., 2011; Qualmann et al., 1999). To test if Dyn2 was recruited to caveolae directly by EHD2 or Pac2, these proteins were depleted in Dyn2-GFP-Cav1-mCh FlpIn cells and individual caveolae were photobleached (Fig. 3 B, Fig. 1 C, and Fig. S3 C). The FRAP revealed no change in recovery rate of Dyn2-GFP at caveolae in the absence of EHD2 or Pac2 as compared with ctrl cells (Fig. 3 B). These results show that Dyn2 is not directly dependent on EHD2 nor Pac2 for its recruitment to caveolae. There was also no significant indirect effect on the localization of Dyn2 to caveolae in the EHD2- or Pac2-depleted cells (% colocalization 40.4 ± 2.93 and 38.8 ± 2.94, respectively) as determined by quantification of TIRF images.

### Dyn2 and EHD2 cooperatively stabilize caveolae to the PM

When comparing the surface duration of caveolae in EHD2-Cav1-mCh HeLa FlpIn cell lines, we confirmed that like Dyn2, the extra EHD2 aids in caveola stabilization (Fig. 3 C; Mohan et al., 2015). Interestingly, however, while caveola displacement was not altered in Dyn2-Cav1-mCh cells, the displacement was significantly shorter in EHD2-Cav1-mCh cells as compared with ctrl cells (Fig. 3 C and Fig. S3 E). This shows that EHD2, but not Dyn2, influences the lateral movement of caveolae. As Dyn2, similarly to EHD2, confined caveolae to the cell surface, we wanted to compare the localization and function of these proteins at caveolae. To address if Dyn2 and EHD2 could localize to the same caveola, BFP-tagged EHD2 was overexpressed in induced Dyn-GFP-Cav1-mCh HeLa FlpIn cells followed by TIRF imaging (Fig. 3 D). We found that Dyn2 localized to 72 ± 3.2% of the EHD2-positive caveolae and 93 ± 2.41% of Dyn2-positive caveolae were also positive for EHD2 (Fig. 3 B). Dyn2 spots devoid of Cav1-mCh were always free from EHD2-BFP. Immunogold colabeling of endogenous Dyn2 and EHD2 in 3T3-L1 cells verified that all caveolae positive for Dyn2 were also positive for EHD2, showing that recruitment of these proteins is not mutually exclusive (Fig. 3 E). Yet, quantification of immunogold labeling showed that EHD2 labeling was present on the great majority of caveolae (>95% mean gold per caveolae, 3.2 ± 2.1), while Dyn2 labeling was only observed on a subset of caveolae (1.9%, mean gold, 2.35 ± 2.5). All CCPs were positive for dynamin under these conditions (mean gold per CCP, 31.2 ± 4.1; Fig. 3 E). The difference in the estimated percentage of Dyn2-positive caveolae when comparing endogenous Dyn2 in fixed cells to fluorescently tagged Dyn2 in live cells suggests that caveola association of Dyn2 is sensitive to methodological differences and/or might vary between cell types. However, our data show that Dyn2 is only localized to a subset of stable caveolae, further implying that Dyn2 and EHD2 might stabilize caveolae via different but cooperative mechanisms.

To further determine if Dyn2 and EHD2 could act cooperatively to stabilize caveolae, we tracked and compared the dynamic track parameters in Dyn-GFP-Cav1-mCh HeLa FlpIn cells following expression of BFP-tagged EHD2. Analysis showed that overexpression of BFP-EHD2 further increased the duration time of caveolae in relation to the already stabilizing effect of Dyn2 in these cells. In agreement with the hypothesis that Dyn2 and EHD2 cooperatively affect caveola stability, depletion of EHD2 from the Dyn2-GFP-Cav1-mCh cells decreased the caveola duration time, but not to the level of EHD2-depleted Cav1-mCh cells (Fig. 3 F and Fig. 1 F). This showed that Dyn2 still has a stabilizing effect even following EHD2 depletion suggesting that the proteins might act via distinct mechanisms.

### Dyn2 localizes to the caveolae bulb

**Figure 4.**
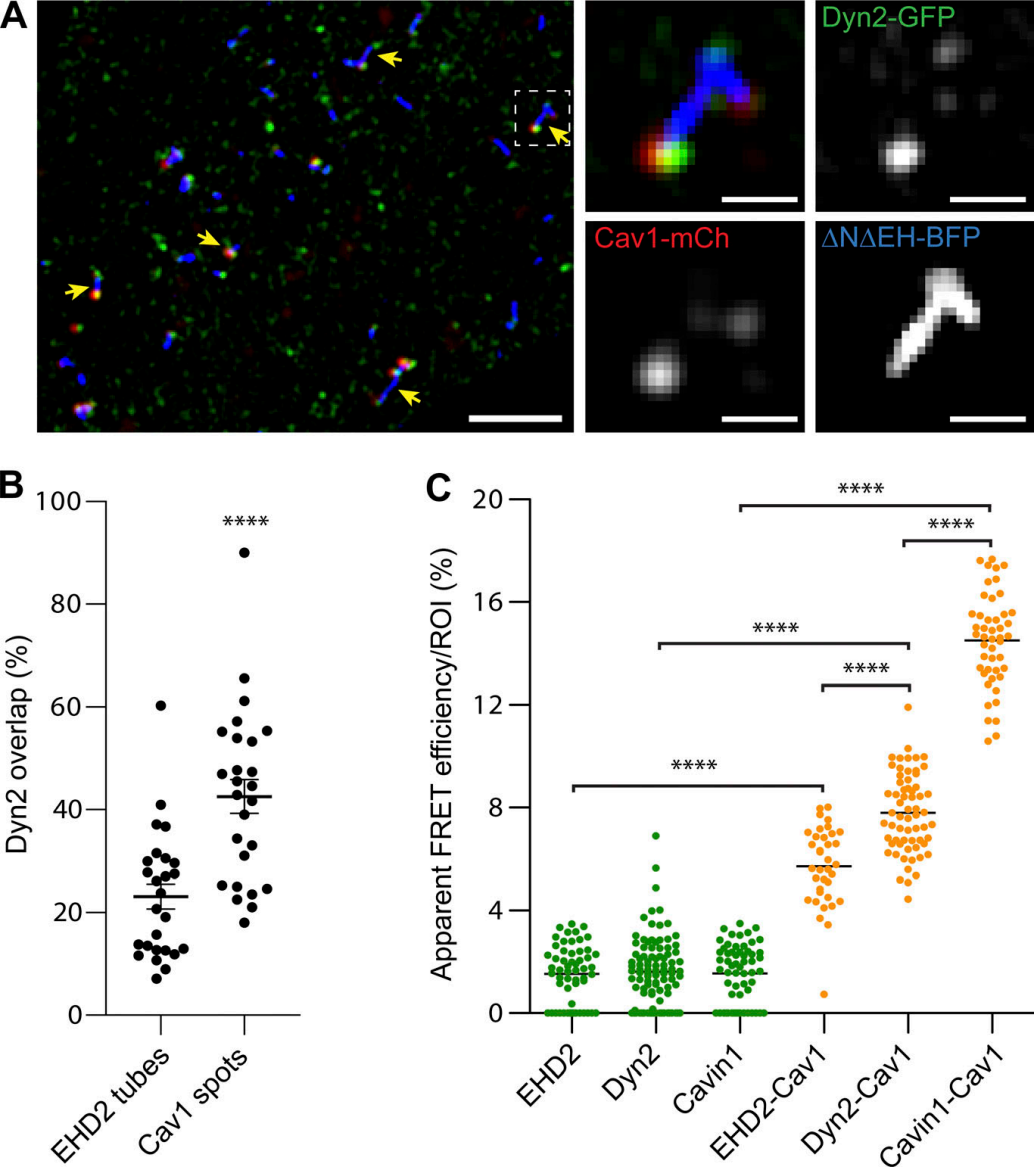
**Dyn2-GFP localizes to the bulb and not the neck of caveolae. (A)** SIM image of a Dyn2-GFP-Cav1-mCh cell transiently expressing ΔNΔEH EHD2-BFP. Yellow arrows highlight Dyn2-GFP localization to Cav1-mCh at the tip of membrane tubes. Insets show magnification of the indicated area. Scale bar, 2 or 0.4 μm for magnification. The experiment was repeated three times. **(B)** Quantification of the fluorescent area of ΔNΔEH EHD2-BFP–positive tubes or Cav1-mCh–positive spots that were overlapping with Dyn2 fluorescence. Data are shown as scatter dot plot, mean ± SEM from three cells. Significance was assessed using *t* test, ****P ≤ 0.0001. **(C)** Apparent FRET efficiency between GFP and mCh fluorophores in GFP-EHD2-Cav1-mCh cells, Dyn2-GFP-Cav1-mCh cells, or Cav1-mCh transiently expressing Cavin1-GFP. ROIs of structures positive for only GFP (green) or GFP and Cav1 (orange) were used to calculate the apparent FRET efficiency. Significance was assessed using *t* test, ****P ≤ 0.0001.

Our data suggest that EHD2 and Dyn2 work independently at caveolae, yet both proteins are known to oligomerize into rings and helices on highly curved membranes such as the caveolae neck. To decipher whether Dyn2 and EHD2 both were located at the caveolae neck, we expressed the mutant ΔNΔEH-EHD2, known to extend the caveolae neck into long tubules (Hoernke et al., 2017), in the Dyn2-GFP-Cav1-mCh HeLa FlpIn cells (Fig. 4 A). Indeed, using SIM, we detected distinct membrane tubes decorated by ΔNΔEH-EHD2-BFP connected to Cav1-mCh–positive caveolae spots (Fig. 4 A). Interestingly, Dyn2-GFP did not decorate the tubes, but instead colocalized with Cav1-mCh in puncta at the end of the tubes (Fig. 4, A and B), suggesting that Dyn2 localizes to the caveolae bulb.

To further address the interactions of caveola components in live cells, we used fluorescence-life-time microscopy (FLIM). This methodology is based on Förster resonance energy transfer (FRET) energy transfer between fluorophores in close proximity affecting the fluorescence lifetime. First, the lifetime of singly expressed Dyn2-GFP, GFP-EHD2, or Cavin1-GFP was determined (Fig. 4 B and Fig. S3 F). Next, FLIM was measured on structures where Cav1-mCh colocalized together with either Dyn2-GFP, GFP-EHD2, or Cavin1-GFP, and the apparent FRET efficiency was calculated (Fig. 4 B). Cavin1, which together with Cav1 builds up the caveolae coat, resulted in a very high FRET efficiency. This shows that, as expected, Cavin1 and Cav1 interact in cells. However, EHD2, which localizes at the caveolae neck, showed a low degree of FRET efficiency (Fig. 4 B). Interestingly, the FRET efficiency for Dyn2 was intermediate (Fig. 4 B). These data suggest that Dyn2 and EHD2 are not situated at the same position on caveolae but rather that Dyn2 localizes closer to the caveolae bulb.

### Dyn2 oligomerization and stimulated GTPase activity are not required for stabilizing caveolae at the PM

**Figure S4.**
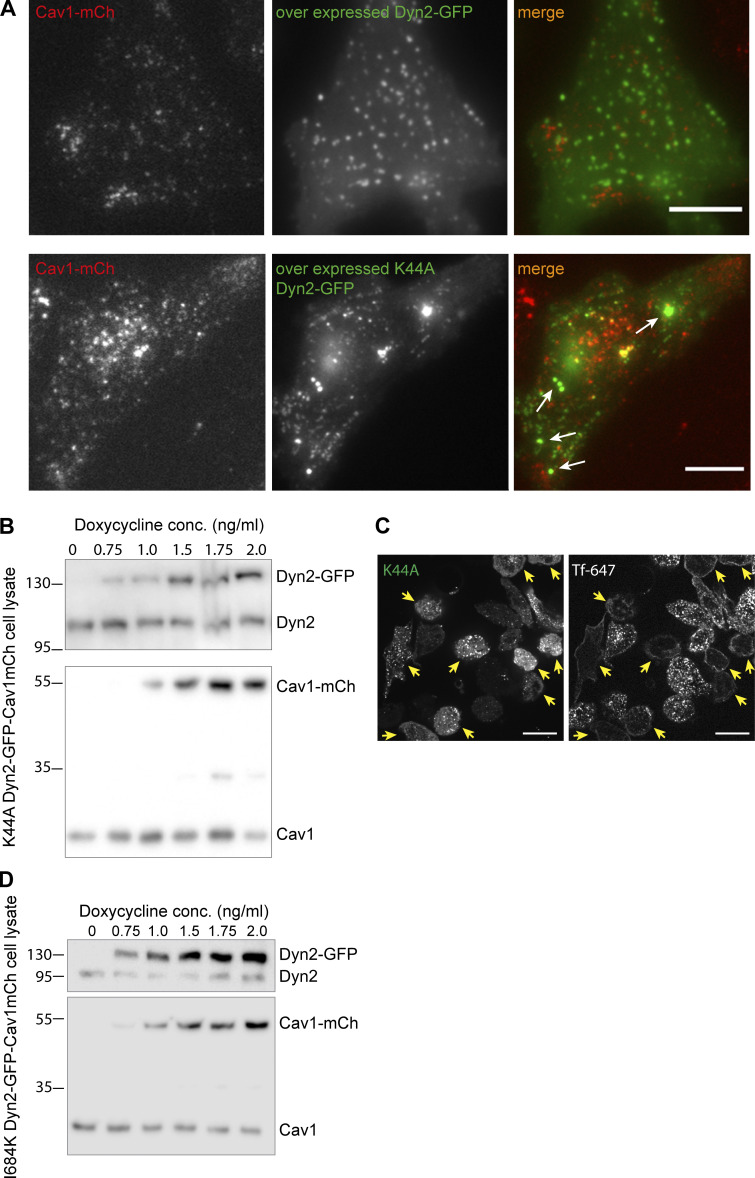
**Overexpression of Dyn2-GFP and K44A Dyn2-GFP, and characterization of protein expression in K44A Dyn2-GFP-Cav1-mCh FlpIn cells and I684 Dyn2-GFP-Cav1-mCh FlpIn cells. (A)**Representative images of Cav1-mCh HeLa FlpIn cells transiently expressing wild-type Dyn2-GFP (top panel) or K44A Dyn2-GFP (bottom panel). Scale bar, 10 μm. **(B)** Immunoblot of K44A Dyn2-GFP-Cav1-mCh cells treated with concentrations of doxycycline as indicated. Doxycycline concentration used for experiments were 1.0 ng/ml. Molecular weights in kD. **(C)** Representative images of fixed K44A Dyn2-GFP Cav1-mCh FlpIn cells incubated for 10 min with Tf-647 (5 μg/ml) at 37°C. Yellow arrows indicate cells with high expression of K44A Dyn2-GFP and the coinciding lack of Tf-647 uptake. Scale bar, 10 μm. **(D)** Immunoblot of I684K Dyn2-GFP-Cav1-mCh cells treated with concentrations of doxycycline as indicated. Molecular weights in kD. Source data are available for this figure: SourceData FS4.

**Figure 5.**
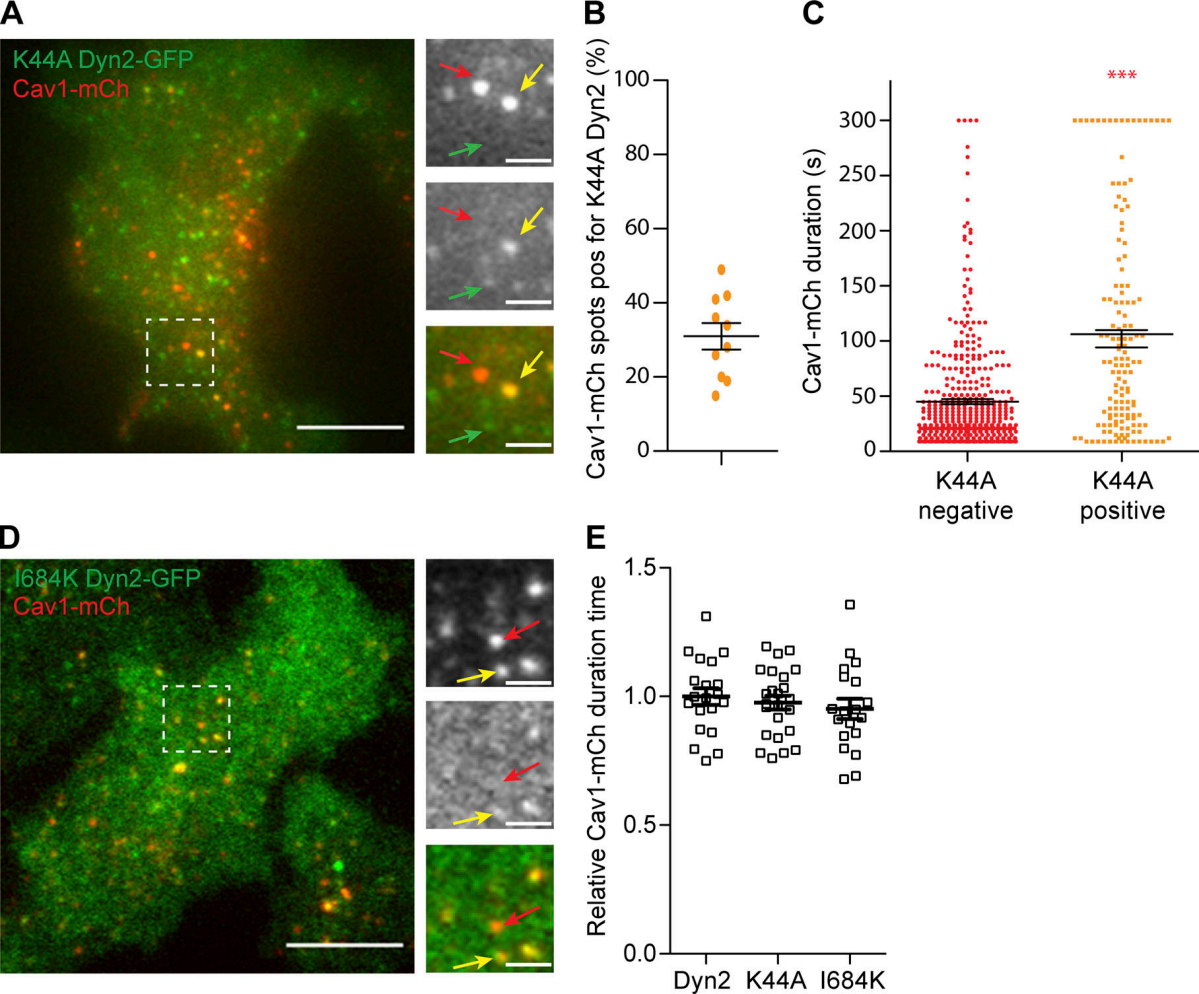
**Dyn2 mutants defective in oligomerization or stimulated GTPase activity localizes to and stabilizes caveolae to the PM. (A)** Representative image from TIRF movie of K44A Dyn2-GFP-Cav1-mCh cell. Red arrow highlights structure only positive for Cav1-mCh, yellow arrow highlights structure positive for both Cav1-mCh and K44A Dyn2-GFP, and green arrow highlights structures only positive for K44A Dyn2-GFP. Scale bar, 10 or 2 μm for magnification. **(B)** Quantification of percentage of caveolae positive for K44A Dyn2-GFP. Data from 10 cells are shown as scatter dot plot, mean ± SEM. **(C)** Caveolae track duration times and displacement lengths divided in pools of K44A Dyn2-GFP–negative (red) or –positive (orange). K44A Dyn2-GFP-Cav1-mCh cells were imaged on TIRF over 5 min. All Cav1-mCh spots were followed and scored whether they were positive or negative for Dyn2-GFP. Graph shows all Cav1-mCh tracks from three different cells. Analysis was performed using Imaris software, and data are presented as scatter dot plot, mean ± SEM. Significance was assessed using *t* test. **(D)** Representative image from TIRF movie of I684K Dyn2-GFP-Cav1-mCh cell. Red arrow highlights structure only positive for Cav1-mCh, yellow arrow highlights structure positive for both Cav1-mCh and I684K. Scale bar, 10 or 2 μm for magnification. **(E)** Quantification of Cav1-mCh track duration time in Dyn2-GFP-Cav1-mCh cells (Dyn2), K44A Dyn2-GFP-Cav1-mCh cells (K44A), and I684K Dyn2-GFP-Cav1-mCh cells (I684K) following 72 h depletion of endogenous Dyn2. Numbers were related to Dyn2-GFP-Cav1-mCh cells. Mean ± SEM from at least 19 cells per condition is shown. Significance was assessed using *t* test, ***P ≤ 0.001.

Dynamin has been shown to function at the bulb of maturing CCPs (Aguet et al., 2013; Reis et al., 2015) where self-assembly and stimulated GTPase activity were not required (Sever et al., 2000; Sever et al., 1999). Given that Dyn2 localized to the caveolae bulb and stabilized caveolae independently of EHD2, we further investigated if the intrinsic activities of Dyn2 were important. To test if the GTP cycle of Dyn2 influenced its role at caveolae, we employed the commonly used transition-state-deficient dynamin mutant K44A (Dyn2 K44A). This mutant has been shown to oligomerize but to have decreased GTP affinity and hydrolysis rate, and its expression in cells inhibits fission of clathrin-coated vesicles (Damke et al., 1994). It should be noted that transiently overexpressed Dyn2, as well as Dyn2 K44A, mislocalizes to stable aggregates (Fig. S4 A). Therefore, we constructed inducible K44A-Dyn2-GFP-Cav1-mCh HeLa FlpIn cells (Fig. 5 A and Fig. S4 B). In agreement with previous work, we detected a clear dose-dependent inhibition of Tf uptake following expression of this mutant (Fig. S4 C). Cells imaged by TIRF revealed that Dyn2 K44A also localized to caveolae, although the percentage of Cav1-mCh spots colocalizing with dynamin was lower than that of wild-type dynamin (Fig. 5, A and B). Therefore, to be able to compare the effects on caveolae stability, caveolae were divided into pools positive versus negative for K44A Dyn2-GFP. Tracking of the caveolae dynamics showed that the presence of Dyn2 K44A increased the Cav1-mCh track duration time to a similar extent as to that of wild-type dynamin (Fig. 5 C and Fig. 2 G). This showed that GTP hydrolysis was not required for the ability of Dyn2 to stabilize caveolae at the PM.

To address if oligomerization of Dyn2 was required for stabilization of caveolae, we generated inducible I684K-Dyn2-GFP-Cav1-mCh HeLa FlpIn cells (Fig. 5 D and Fig. S4 D). This dynamin mutant has been shown to abolish oligomerization and inhibit Tf uptake and microtubule stability when overexpressed (Guo et al., 2022; Song et al., 2004). TIRF imaging showed that Dyn2 I684K was localized to the entire PM and that the distinct punctuate stain characteristic of Dyn2 assemblies was less clear (Fig. 5 D). Dyn2 I684K fluorescence was slightly enriched at a subfraction of the caveolae. Yet, the difference in this signal as compared to the overall PM was too small to allow for reliable quantification and subdivision into Dyn2-positive and -negative caveolae. Therefore, to test if oligomerization deficient Dyn2 influenced caveolae dynamics, we compared the mean track duration in FlpIn cells expressing Dyn2-GFP, I684K Dyn2-GFP, and K44A Dyn2-GFP. We removed the contribution of endogenous Dyn2 in these cells by siRNA-specific depletion. In Dyn2-GFP expressing cells, there was no significant difference in the duration time of caveolae following depletion of endogenous Dyn2 as compared with ctrl siRNA-treated cells. This suggested that Dyn2-GFP could rescue the effect of Dyn2 depletion in agreement with previous results. Similarly, there were only minor decreases in the duration time in I684K and K44A expressing cells, suggesting that also these Dyn2 mutants can substitute for endogenous Dyn2. Taken together, our data show that similar to the proposed role of Dyn2 in early stages of CCV formation, the stabilizing role of Dyn2 at caveolae does not require oligomerization and stimulated GTPase activity.

### Dyngo4a immobilizes caveolae at the PM independently of Dyn2

**Figure S5.**
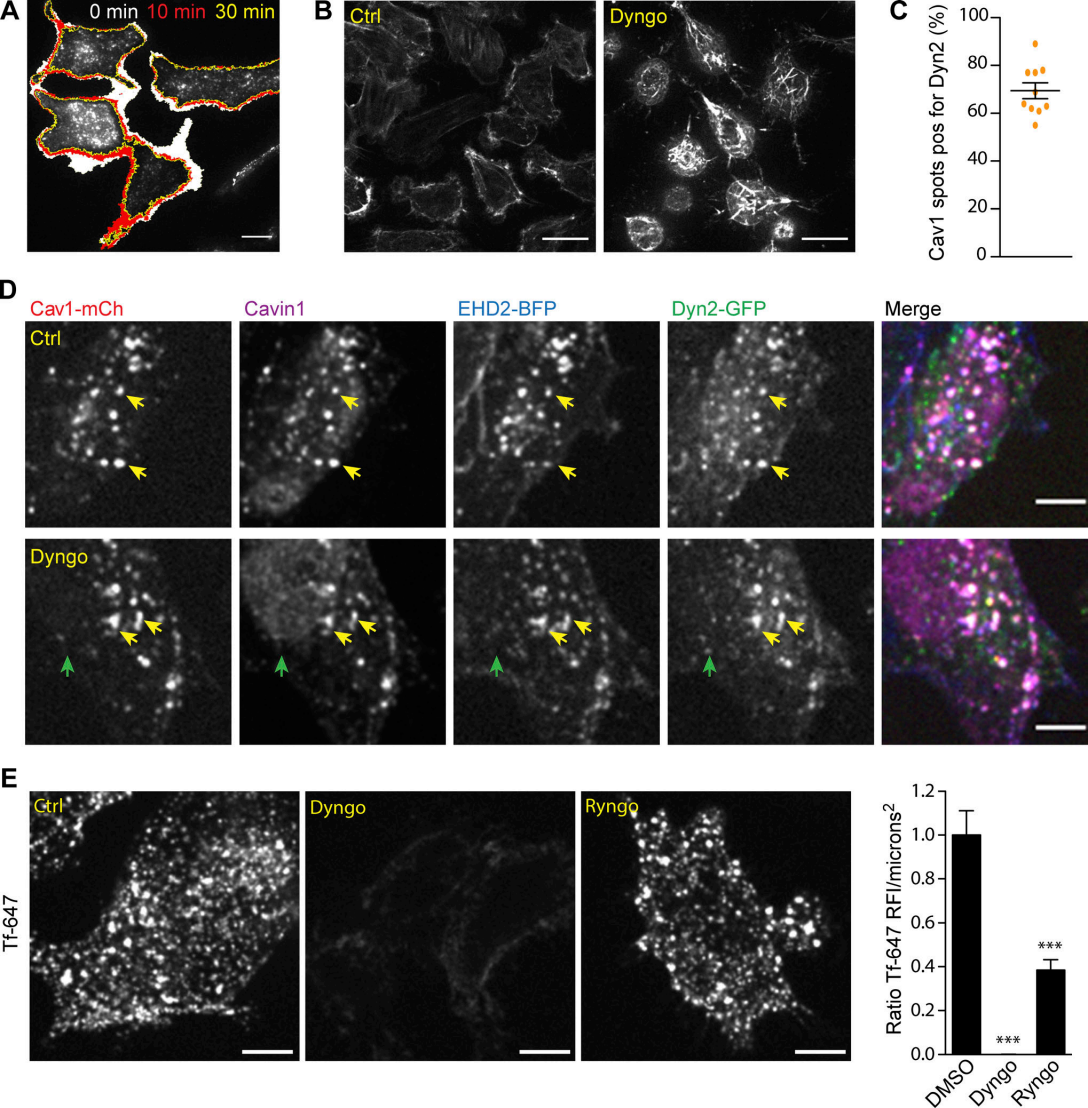
**Dyngo 4a treatment affects cell morphology and F-actin. (A)** Example of basal PM retraction after Dyngo 4a addition. White area depicts the area retracted between time point 0 to 10 min. Red area depicts the area retracted between time point 10 to 30 min. Yellow outline illustrates the basal PM surface area after 30 min treatment with Dyngo 4a. Scale bar, 10 μm. **(B)** Representative confocal image of basal membrane in Dyn2-GFP-Cav1-mCh cells treated with DMSO (Ctrl) or Dyngo 4a for 30 min and incubated with the F-actin marker SiR-actin. Scale bar, 20 μm. **(C)** Quantification of percentage of caveolae positive for Dyn2-GFP after 30 min Dyngo 4a treatment. Data are shown as scatter dot plot, mean ± SEM. **(D)** Representative immunofluorescent staining of Dyn2-GFP-Cav1-mCh cells. Cells transiently expressing EHD2-BFP were treated with DMSO (Ctrl) or Dyngo 4a for 30 min and then fixed and stained with cavin1 antibody (magenta). Yellow arrows depict colocalizing structures and green arrow depicts only Dyn2-GFP–positive structure. Scale bar, 10 μm. **(E)** Representative image of fixed Dyn2-GFP-Cav1-mCh cells treated with DMSO (Ctrl), Dyngo 4a, or Ryngo before 10 min incubation with Alexa fluor 647 conjugated Tf (5 μg/ml) at 37°C. Scale bar, 10 μm. Quantification show relative Tf-647 fluorescent intensity/microns^2^. Significance was assessed using *t* test, ***P ≤ 0.001.

**Figure 6.**
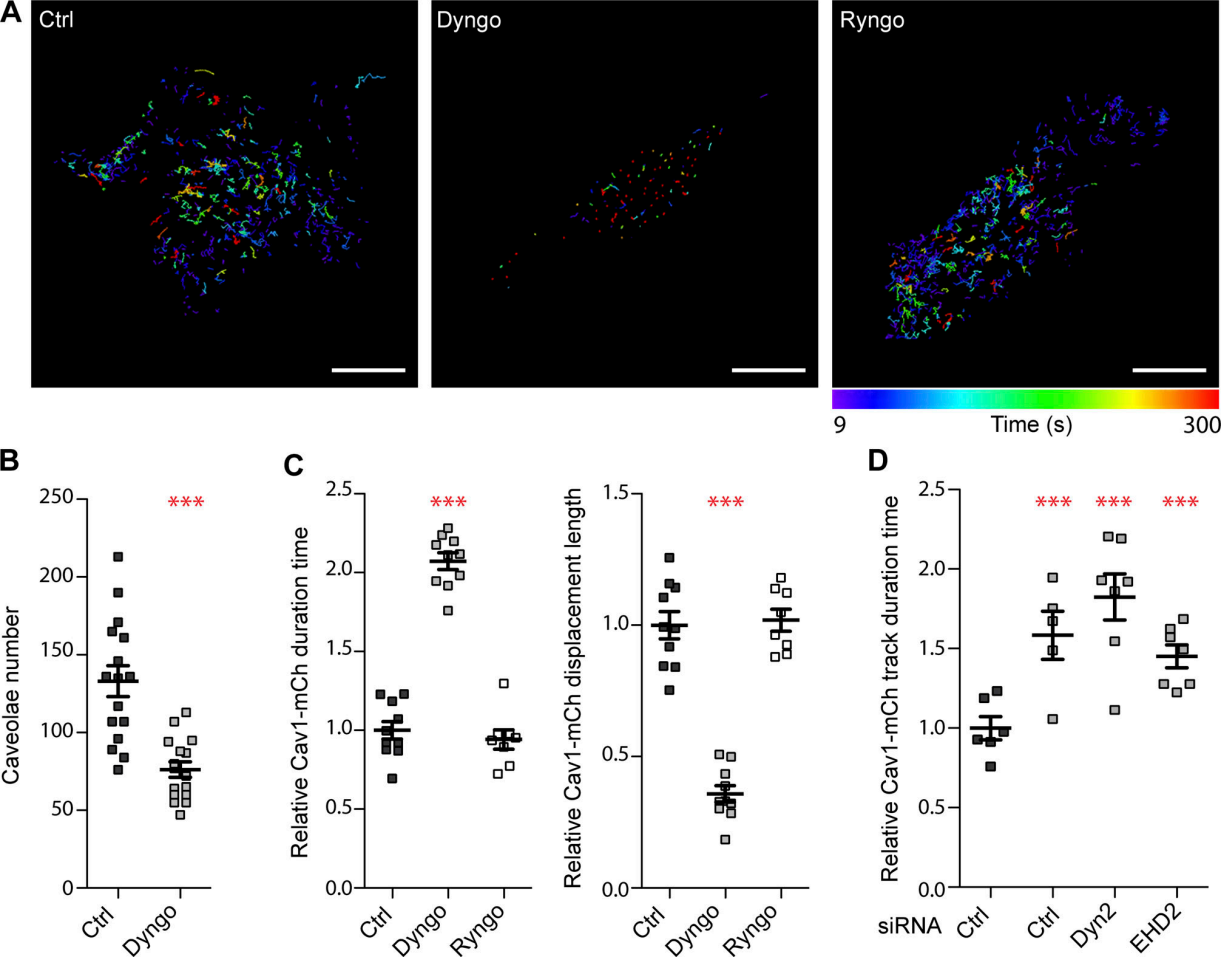
**The stabilization of caveolae by Dyngo 4a is independent of Dyn2. (A)** Color-coded trajectories of caveolae in Dyn2-GFP-Cav1-mCh cells pretreated with DMSO (Ctrl), Dyngo 4a, or Ryngo 1-23 for 30 min before imaged on TIRF over 5 min. Scale bar, 10 μm. **(B)** Quantification of percentage of caveolae positive for Dyn2-GFP after 30 min treatment with DMSO (black) or Dyngo 4A (gray). Data from at least 16 cells per condition are shown as scatter dot plot, mean ± SEM. **(C)** Quantification of Cav1-mCh track duration time and displacement length in cells as indicated. Numbers were related to ctrl-treated cells. Analysis was performed using Imaris software, and track mean from at least eight cells per condition are shown ± SEM. **(D)** Quantification of Cav1-mCh track duration time in Cav1-mCh cells treated with siRNA as indicated prior to pretreatment of DMSO (Ctrl) or Dyngo 4a as in A. Numbers were related to DMSO-treated ctrl siRNA-treated cells (black). Analysis was performed using Imaris software, and track duration time from at least eight cells per condition is shown as ± SEM. Significance was assessed using *t* test, ***P ≤ 0.001.

The small molecule dynamin inhibitor Dyngo 4a has been shown to prevent membrane-stimulated GTPase activity of dynamin and halt CCV endocytosis (McCluskey et al., 2013). Since Dyngo 4a has been used to block caveola fission, we wanted to investigate the effect of Dyngo 4a on caveola dynamics. Dyngo 4a was added to Dyn-GFP-Cav1-mCh HeLa FlpIn cells and incubated for 30 min followed by TIRF imaging. As previously demonstrated, cells retracted in response to Dyngo 4a treatment (Park et al., 2013), reducing the basal surface area (Fig. S5 A). Indeed, Dyngo 4a was reported to have off-target effects on fluid-phase endocytosis and the actin cytoskeleton, and we could confirm that the F-actin was disrupted in cells treated with Dyngo 4a as compared with mock-treated cells (Fig. S5 B). The number of Cav1-mCh spots at the basal surface was also dramatically reduced (Fig. 6, A and B), but around 70% of the remaining spots were positive for Dyn2 (Fig. S5 C). We could confirm that the spots were surface-connected caveolae as they were positive for Cavin1 and EHD2 (Fig. S5 D). The increased ratio of caveolae positive for Dyn2 could suggest that these caveolae were more resistant to Dyngo 4a treatment. As previously shown, CCV-mediated uptake of Tf was blocked in Dyngo 4a–treated cells (Fig. S5 E).

Tracking of Cav1-mCh spots in Dyngo 4a–treated cells revealed a dramatic effect on the caveola dynamics (Fig. 6, A and C, and Video 5). The track duration times were increased to the double and the track displacement length had decreased to less than half of that of ctrl-treated cells (Fig. 6, A and C). These data revealed that caveolae had become almost static in comparison to mock-treated cells (Fig. 6 A). This was surprising since these effects on caveola dynamics were several times higher than the stabilizing effect of Dyn2 or Dyn2 K44A or the increased mobility observed in cells depleted of Dyn2 or EHD2. To determine if the dramatic effects were indeed due to inhibition of dynamin GTPase activity, the Dyngo 4a treatment was repeated in Cav1-mCh FlpIn HeLa cells depleted of Dyn2. Surprisingly, the drug had the same stabilizing effect on the caveolae in these cells (Fig. 6 D). Similarly, EHD2 KD did not significantly influence the stabilizing effect of Dyngo 4a. These data showed that caveolae can indeed be immobilized using Dyngo 4a as previously proposed, but that this effect is not due to block of Dyn2 GTPase activity.

**Video 5.** **Dyngo 4a treatment results in static caveolae within the PM.** Representative live cell video of Dyn2-GFP-Cav1-mCh FlpIn cells treated with Dyngo 4a for 30 min prior to imaging. Cells were imaged on TIRF every third s for 5 min. Cav1-mCh is seen in red and Dyn2-GFP in green. Frame rate: 10 frames per second.

In view of these results, which go against a long-standing dogma that has developed in the field, we wanted to test if stimulation of the actin-dependent oligomerization of Dyn2 into rings influenced its stabilizing effect on caveola dynamics. For this, we used Ryngo 1-23, which has been reported to stimulate dynamin GTPase activity by actin-dependent oligomerization (Gu et al., 2014). This dynamin modulator has been shown to induce actin filament rearrangements (Lees et al., 2015). As previously shown (Sandgren et al., 2010), Ryngo 1-23 treatment impaired the uptake of Tf in cells (Fig. S5 E). Analysis of the dynamic track parameters of caveolae showed that Ryngo 1-23 had no apparent effect on the caveolae track duration time or displacement length as compared with ctrl-treated cells (Fig. 6, A and C). Taken together, our results show that stimulation or inhibition of the GTPase activity of Dyn2 does not influence caveola dynamics, but that Dyngo 4a has dramatic, Dyn2-independent effects on caveola stability.

### Lipid-induced fission of caveolae is dynamin independent

**Figure 7.**
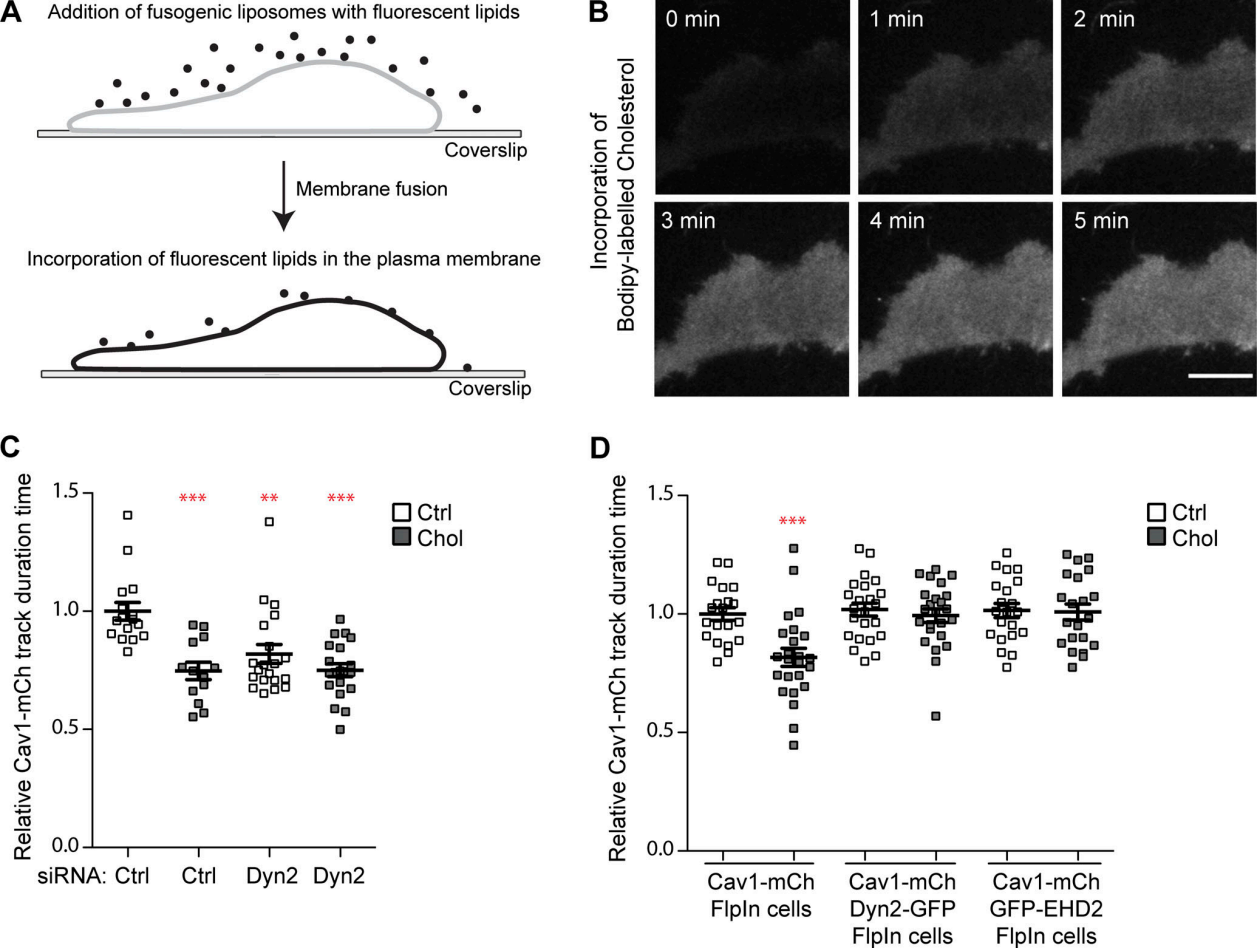
**Lipid induced fission of caveolae is counteracted by Dyn2-GFP. (A)** Schematic of fusogenic liposome methodology. **(B)** Time series showing the incorporation of Bodipy-labeled cholesterol into the PM of a Cav1-mCh cell. Scale bar, 10 μm. **(C)** Quantification of track duration time in Cav1-mCh cells pre-treated with Ctrl or Dyn2 siRNA as indicated. Cells were non-treated (white) or treated with fusogenic liposomes containing cholesterol (black) for 20 min prior to imaging. Analyses were performed using Imaris software, and track mean from at least 13 cells per condition are shown ± SEM. **(D)** Quantification of track duration time in Cav1-mCh cells, Dyn2-GFP-Cav1-mCh cells, or GFP-EHD2-Cav1-mCh cells non-treated (white) or treated with fusogenic liposomes containing cholesterol (black) for 20 min prior to imaging. Analysis was performed using Imaris software, and track mean from at least 20 cells per condition are shown ± SEM. Significance was assessed using *t* test, **P ≤ 0.01, ***P ≤ 0.001.

Increased levels of cholesterol in the PM have been shown to drive fission of caveolae (Hubert et al., 2020b). To address if Dyn2 was involved in this process, we used the same methodology involving fusogenic liposomes to rapidly incorporate cholesterol in the PM (Fig. 7, A and B). Real-time TIRF imaging showed that the PM levels of fluorescent cholesterol indeed increased over time (Fig. 7 B). Cav1-mCh cells transfected with ctrl or Dyn2 siRNA were treated with fusogenic liposomes containing unlabeled cholesterol for 15 min before being imaged on TIRF. As expected, tracking analysis showed that cholesterol addition to Cav1-mCh cells significantly decreased the time that the caveolae spent at the PM compared with non-treated cells (Fig. 7 C). Interestingly, the duration time in cells depleted of Dyn2 was also reduced by cholesterol addition to the same level as in cells treated with ctrl siRNA (Fig. 7 C). This revealed that Dyn2 was not required for cholesterol-driven fission of caveolae.

To test if Dyn2 instead could restrain the lipid-driven fission of caveolae, the effects of cholesterol incorporation on caveola dynamics in Dyn2-GFP-Cav1-mCh cells was monitored and compared to the dynamics in Cav1-mCh cells. Interestingly, cholesterol administration had no significant effect on the caveola track duration in Dyn2-GFP-Cav1-mCh cells (Fig. 7 D), showing that a slight excess of Dyn2 was enough to restrain the lipid-driven fission. Similarly, the elevated levels of EHD2 in GFP-EHD2-Cav1-mCh FlpIn cells also prevented cholesterol-induced fission (Fig. 7 D), as previously shown (Hubert et al., 2020b). These data show that dynamin, just like EHD2, prevents caveola-fission induced by cholesterol sequestering, and furthermore, that lipid-induced fission of caveolae from the PM is dynamin independent. Taken together, our results show that Dyn2 significantly contributes to the atypical dynamics of caveolae by stabilizing the PM association of these specialized membrane domains.

## Discussion

Caveolae display atypical dynamics in the sense that such invaginations are stably associated with the PM over time. Yet, the mechanisms controlling the stability and fission of the caveola neck are not fully understood. Herein, we have studied the role of Dyn2 on caveola dynamics in living cells using TIRF microscopy and single particle tracking. In contrast to its role in CCV fission, we find that Dyn2 acts as a stabilizing protein, increasing the time caveolae spend at the PM. Dyn2 was detected on a subfraction of caveolae, and these Dyn2-positive caveolae displayed PM duration times that were twice as long as the caveolae devoid of Dyn2. Depletion of Dyn2 from cells destabilized the PM association of caveolae leading to shorter duration times. Furthermore, Dyn2 was not required for lipid-induced caveola fission as triggered by exogenous addition of cholesterol. However, excess levels of Dyn2 could counteract such lipid-induced fission. This stabilizing role of Dyn2 did not require GTP-hydrolysis or oligomerization, and Dyn2 localized to the bulb of caveolae rather than the neck region. Based on our data, we find that Dyn2 is not required for caveola formation or fission. We propose that it acts cooperatively with EHD2, but via an independent mechanism, to restrain a subset of caveolae to the PM.

Our results, which go against the current dogma on caveola fission (Matthaeus and Taraska, 2021), can be reconciled with previous data and build a refined model for how Dyn2 influences caveola dynamics. Contrary to our findings, dynamin and GTP-hydrolysis were first proposed to potentiate fission of caveolae based on disappearance of Cav1 from the pellet of purified PMs following 1 h incubation with cytosol and GTP (Oh et al., 1998; Schnitzer et al., 1996). Yet, it was not shown that the loss of Cav1 from the pellet was a result of specific fission and release of caveolae per se. Cav1 has been shown to be released from the PM by alternative vesicles in the absence of the caveola coat machinery (Chaudhary et al., 2014). Furthermore, although Dyn2 could drive caveola fission in this simplified biochemical system, the role in an intact cell might be completely different. Additionally, microinjection of antibodies directed against Dyn2 in hepatocytes decreased the uptake of cholera toxin, which was interpreted to indicate that the internalization of caveolae had been blocked (Henley et al., 1998). However, cholera toxin is not a specific marker of caveolae and has been shown to be mainly internalized via other clathrin-independent mechanisms as reviewed in Kenworthy et al. (2021). This demonstrates the need for direct analysis of caveola dynamics in living cells. Here, we have used inducible cell lines with expression levels of caveolin and dynamin close to endogenous levels in combination with TIRF microscopy and single-particle tracking of caveolae to directly determine the PM duration and mobility of a large number of caveolae under different conditions. The combined analysis of caveola dynamics following Dyn2 depletion, expression of wild-type and specific Dyn2 mutants, inhibitory drugs, and lipid-induced fission of caveolae, clearly show that Dyn2 is not required for fission. A number of studies have used overexpression of Dyn2 mutants such as K44A or treatment with drugs such as Dynasore or Dyngo 4a to restrict caveola budding. These data are consistent with our data, but we propose that this is not due to inhibition of Dyn2 but rather the intrinsic ability of Dyn2 to stabilize caveolae and off-target effects of the drugs, respectively.

In further support of the conclusion that Dyn2 does not perform fission of caveolae, our data show that Dyn2 firmly is associated with caveolae over time and not accumulated prior to fission. This pattern of Dyn2 localization differs from that seen for CCVs, where a spike in Dyn2 recruitment is followed by CCV fission (Merrifield et al., 2002). For Dyn2 to play a direct role in caveola fission, it would be predicted to assemble at the caveolae neck. Yet, by extending the caveolae neck into tubes using the ΔNΔEH-EHD2 mutant, we found that Dyn2 was not enriched at such tubules but rather localized closer to the bulb together with Cav1. FLIM-FRET analysis verified that Dyn2 was in closer proximity to Cav1 than to EHD2. These data are in agreement with previous results suggesting that Dyn2 and Cav1 interact directly (Yao et al., 2005). The detection of a sub-stochiometric interaction between Dyn2 and Cav1 vesicles (10–15 Dyn2 molecules per 100–150 Cav1) in a cell-free system further supports direct interaction at the caveolae bulb (Jung et al., 2018). Furthermore, FRAP analysis showed that GTPase-triggered disassembly of Dyn2 at caveolae is slow. This implies that stimulated GTP-hydrolysis might not be required for stabilization of caveolae at the PM. Indeed, the GTP hydrolysis deficient mutant K44A stabilized PM association of caveolae similar to that seen with wild-type Dyn2, although this mutant inhibits fission of CCV. This showed that GTP hydrolysis by Dyn2 is not required for caveola stabilization, which is reasonable since GTP-hydrolysis is known to facilitate membrane tubule constriction. Similarly, the Ryngo 1-23 inhibitor, which is reported to promote actin-dependent oligomerization and GTP hydrolysis by dynamin, inhibited CCV-mediated uptake of Tf but had no significant effect on caveola dynamics. This suggests that stimulation of the GTPase activity coupled with actin-dependent oligomerization of Dyn2 was not important for caveola stabilization. Furthermore, analysis of caveola dynamics following expression of the assembly-deficient mutant Dyn2 I684K suggested that oligomerization is not required for caveola stabilization. In summary, our findings suggest that Dyn2 localizes to the caveolae bulb and stabilizes caveolae via a mechanism that does not require GTP hydrolysis or oligomerization, distinct from the role of dynamin during fission and release of CCVs. Further work is necessary to understand the still mechanistically mysterious regulatory roles of dynamin both in caveolae and CCV formation.

Dyn2 has been previously shown to localize to caveolae (Oh et al., 1998) in agreement with our findings. Yet, the amount of caveolae that are positive for Dyn2 is difficult to measure and could vary between cell types and the techniques used. In live Dyn2-GFP-Cav1-mCh cells, we found that ∼40% of the caveolae were positive for dynamin, but these cells have elevated levels of Dyn2. Immunogold labeling of endogenous Dyn2 in 3T3L1 cells suggested that rather a small fraction of caveolae were positive for Dyn2, but this could be underestimating the amount based on fixation and poor antibody binding. In addition to Dyn2, caveola stabilization has been shown to depend on peripheral membrane proteins such as EHD2 and Pac2. Although dynamins have been suggested to interact with both EHDs and pacsins (Jakobsson et al., 2011; Qualmann et al., 1999), we found that Dyn2 is not recruited to caveolae directly by EHD2 or Pac2. We show that while EHD2 is present at the majority of caveolae, Dyn2 and Pac2 are limited to a subset. Yet, immunogold labeling and TIRF microscopy revealed that Dyn2 and EHD2 could be simultaneously detected on the same caveolae. Taken together, all data supports the conclusion that a distinct pool of caveolae is positive for Dyn2, but the precise size and the potentially unique properties of this caveolae pool are currently not known. Based on these data, we propose that Dyn2 should not be considered a core component of caveolae such as cav1, cavin1, and EHD2, but rather an accessory regulatory protein.

Although both Dyn2 and EHD2 stabilize caveolae, our data suggest that they act via different mechanisms. Analysis showed that overexpression of EHD2-BFP in Dyn2-GFP-Cav1-mCh cells further increased the duration time of caveolae in relation to the already stabilizing effect of Dyn2 in these cells. Similarly, depletion of EHD2 from the Dyn2-GFP-Cav1-mCh cells decreased the caveola duration time, but not to the level of Cav1-mCh cells (Fig. 3 F and Fig. 1 F). Furthermore, depletion of Dyn2 or EHD2 in Cav1-mCh cells both resulted in decreased caveola duration time. However, while depletion of EHD2 led to longer caveola displacement length, this parameter was unaffected in Dyn2-depleted cells. Likewise, increasing the levels of Dyn2 or EHD2 in the inducible cell lines resulted in caveola stabilization. However, the shorter displacement length found in GFP-EHD2-Cav1-mCh expressing cells could not be observed in Dyn2-GFP-Cav1-mCh cells. Mobility of caveolae in the plane of the membrane has been shown to depend on F-actin (Thomsen et al., 2002), and caveolae are frequently found to align with actin filaments. Coupling of caveolae to F-actin via Filamin A (Stahlhut and van Deurs, 2000), an actin-binding protein, was found to increase internal trafficking of caveolae (Sverdlov et al., 2009). Moreover, both EHD2 and Dyn2 have been suggested to interact with actin and regulate actin polymerization (Gu et al., 2010; Zhang et al., 2020). It is possible that both Dyn2 and EHD2 stabilize the PM association of caveolae, but that they influence actin coupling and polymerization differently, which affects caveola displacement in the membrane.

Our data support a direct role of Dyn2 in caveola stabilization. Yet, effects of Dyn2 inhibition and depletion on other processes such as actin polymerization and CCV endocytosis might also indirectly affect caveola dynamics. This might influence the overall membrane tension and thereby caveolae, which is known to respond to alterations in tension. Interestingly, treatment of cells with Dyngo 4a resulted in a dramatic increase in caveola stability. However, this impact remained even if Dyn2 was depleted from the cells, showing that the effect was not Dyn2 dependent. Furthermore, we demonstrate that treatment with this small dynamin effector modulator disrupted the F-actin severely and affected cell morphology in agreement with previous work showing that Dyngo 4a has off-target effects (Park et al., 2013). Instead, we propose that Dyngo 4a treatment generally affects PM properties, thereby dramatically impacting caveola dynamics. Interestingly, Dynasore (a dynamin inhibitor closely related to Dyngo 4a) has been found to influence the levels and/or accessibility of cholesterol in the PM (Preta et al., 2015). This could heavily influence caveola fission given the proposed key role of cholesterol in this process (Hubert et al., 2020b; Le Lay et al., 2006; Sharma et al., 2004). Although secondary effects of loss of Dyn2 activity could influence caveola dynamics, our data clearly show that Dyn2 is not required for caveola fission. Caveola fission has been shown to be induced by cholesterol or glycosphingolid accumulation, which together with the caveola coat seem to generate an intrinsically unstable domain prone to fission if not balanced by the restraining force of EHD2 (Hubert et al., 2020b). Our data now show that Dyn2 acts independently of EHD2 to also counteract caveola fission. This does not require GTP hydrolysis or oligomerization of Dyn2. We propose that caveola dynamics are delicately balanced by lipid-driven assembly of the caveola coat and restraining forces produced by regulatory proteins such as EHD2 and Dyn2.

## Materials and methods

### Reagents

Dyngo 4a (ab120689) and Ryngo 1-23 (ab146050) were both purchased from Abcam. Human Tf Alexa Fluor 647-conjugate (T23366) was bought from Invitrogen and SiR-actin (SC0001) from Spirochrome. 1,2-dioleoyl-sn-glycero-3-phosphoethanolamine (DOPE), 1,2-dioleoyl-3-trimethylammonium-propane (chloride salt; DOTAP), and TopFluor-cholesterol (Bodipy-Chol) was purchased from Avanti Polar Lipids Inc (Alabaster). DMSO (34943), chloroform (CHCl_3_), and methanol (MeOH) were purchased from Sigma-Aldrich. Rat brain (substansia nigra) protein lysate was a kind gift from Roine El-Habta, Umeå University, Umeå, Sweden.

### Cell lines and constructs

The HeLa Flp-In T-REx Dyn2-EGFP-P2A-Caveolin1-mCherry, EGFP-EHD2-P2A-Caveolin1-mCherry, and Pac2-EGFP-P2A-Caveolin1-mCherry constructs were generated by linearizing pcDNA/FRT/TO/Caveolin1-mCherry (Hubert et al., 2020b) with the restriction enzyme HindIII (Thermo Fisher Scientific). The DNA encoding the EGFP-fusion proteins and the P2A peptide was amplified by PCR and inserted by Gibson assembly using NEBuilder HiFi DNA assembly master mix (New England BioLabs). For creating flanking insert fragments, the following primers were used for Dyn2-EGFP-P2A: 5′-GAC​TCT​AGC​GTT​TAA​ACT​TAA​TGG​GCA​ACC​GCG​GGA​TG-3′ paired with 5′-TTC​CAT​CGA​TCT​TGT​ACA​GCT​CGT​CCA​TGC​C-3′ and 5′-TGG​ACG​AGC​TGT​ACA​AGA​TCG​ATG​GAA​GCG​GAG​CTA​C-3′ paired with 5′-TGG​ATC​CGA​GCT​CGG​TAC​CAC​CTC​TAG​GTC​CAG​GGT​TC-3′. Primers used for Pac2-EGFP-P2A were: 5′-GAC​TCT​AGC​GTT​TAA​ACT​TAA​T GTC​TGT​CAC​CTA​CGA​TG-3′ paired with 5′-TTC​CAT​CGA​TCT​TGT​ACA​GCT​CGT​CCA​TGC​C-3′ and 5′-TGG​ACG​AGC​TGT​ACA​AGA​TCG​ATG​GAA​GCG​GAG​CTA​C-3′ paired with 5′-TGG​ATC​CGA​GCT​CGG​TAC​CAC​CTC​TAG​GTC​CAG​GGT​TC-3′. For creating flanking insert fragments, the following primers were used for EGFP-EHD2-P2A: 5′-GAC​TCT​AGC​GTT​TAA​ACT​TAA​TGG​TGA​GCA​AGG​GCG​AG-3′ paired with 5′-TTC​CAT​CGA​TTT​CAG​CAG​AGC​CCT​TCT​G-3′ and 5′-CTC​TGC​TGA​AAT​CGA​TGG​AAG​CGG​AGC​TAC-3′ paired with 5′-GGA​TCC​GAG​CTC​GGT​ACC-3′. The HeLa Flp-In T-REx K44A Dyn2-EGFP-P2A-Cav1-mCherry construct was obtained by in vitro mutagenesis of the pcDNA/FRT/TO/Dyn2-EGFP-P2A-Cav1-mCherry construct exchanging lysine at position 44 to an alanine using these primers: 5′-CCA​GAG​CGC​CGG​CGCGAG​TTC​GGT​GCT​C-3′ and 5′-GAG​CAC​CGA​ACT​CGCGCC​GGC​GCT​CTG​G-3′. For creation of the HeLa Flp-In T-REx I684K Dyn2-EGFP-P2A-Cav1-mCherry construct the following primers were used to exchange isoleucine at position 684 to a lysine: 5′-GAC​CAT​CAT​GCA​CCT​CAT​GAAGAAC​AAC​ACA​AAG​GCT​TC-3′ and 5′-GAA​GGC​CTT​TGT​GTT​GTTCTTCA​TGA​GGT​GCA​TGA​TGG​TC-3′. The underlining represents the nucleotides in the primer correspond to specific mutations. The Flp-In TRex HeLa cell lines were maintained in DMEM supplemented with 10% (vol/vol) FBS, 100 μg/ml hygromycin B (Thermo Fisher Scientific), and 5 μg/ml blasticidin S HCl (Thermo Fisher Scientific) for plasmid selection at 37°C, 5% CO_2_. Expression at near endogenous levels was induced by incubation with 0.5 ng/ml (Cav1-mCh) and 1.0 ng/ml (EGFP-fusion-P2A-Cav1mCh) doxycycline hyclate (Dox; Sigma-Aldrich) for 16–24 h. All cell lines tested negative for mycoplasma. For generation of the ΔNΔEH EHD2-BFP expression vector, the ΔNΔEH EHD2 mRNA was subcloned from ΔNΔEH EHD2-mCherry construct (Hoernke et al., 2017) using restriction enzymes XhoI and BamHI (Thermo Fisher Scientific) and inserted into the pTagBFP-C (Evrogen) expression vector.

### Fusogenic liposomes

Liposomes were prepared from a lipid mixture of DOPE, DOTAP, and either Bodipy-tagged cholesterol or unlabeled cholesterol at a ratio of 47.5:47.5:5. Lipid blends were in MeOH:CHCl_3_ (1:3, vol/vol). Following the generation of a thin film using a stream of nitrogen gas, the vesicles were formed by addition of 20 mM Hepes (VWR, pH 7.5, final lipid concentration 2.8 μmol/ml) and incubated for 1.5 h at room temperature. Glass beads were added to facilitate rehydration. The liposome dispersion was sonicated for 30 min (Transsonic T 310, Elma).

### Transfections and cell treatments

Cav1-mCh HeLa cells were transfected with Lipofectamine 2000 (Thermo Fisher Scientific) using Opti-MEM I reduced serum medium (Thermo Fisher Scientific) for transient protein expression. For Dyn2, EHD2, Pac2, and Dyn1 depletion, Cav1-mCh HeLa cells were transfected with stealth siRNA, specific against human Dyn1 (#1: 5′-CCG​TAG​ACT​TTG​AGA​AGC​GCA​TTG​A-3′, #2: 5′-GAG​ATC​AGC​TAT​GCT​AT CAAGAATA-3′, #3: 5′-CAT​GGC​CTT​TGA​GAC​CAT​TGT​GAA​A-3′), Dyn2 (5′-CCA​GAT​TCT​TCT​GCT​GAT​CGA​CAT​T-3′), and EHD2 (5′-TTT​CCG​AAA​GGG​TTG​AGT​TTG​CGG​A-3′), all from Thermo Fisher Scientific or ON-TARGETplus siRNA against Pac2 (5′-CAA​ATT​ATG​TGG​AGG​CGA​T-3′; Dharmacon) or scrambled control (Thermo Fisher Scientific) using Lipofectamine 2000 and Opti-MEM according to manufacturer’s instructions. Cells were transfected twice over a period of 72 h before the experiment. Protein levels were analyzed by SDS-PAGE and immunoblotting to Amersham Hybond P 0.45 polyvinylidene difluoride membrane (MERCK) using rabbit anti-Cav1 (ab2910; Abcam), rabbit anti-Dyn1 (ab52661; Abcam), rabbit anti-Dyn2 (PA1-661; Thermo Fisher Scientific), rabbit anti-EHD2, RRID:AB_2833022 (Morén et al., 2012), mouse anti-GAPDH (MAB374; Millipore) rabbit anti-Pac2 (2604), and mouse anti-β-actin (3700), both from Cell Signaling. Following HRP-conjugated secondary antibodies were used: goat anti-rabbit (AS09 602; Agrisera) and goat anti-mouse (A9917; MERCK). In some cases, the membrane was cut after blot before probing with antibodies to simultaneously probe for proteins of different sizes. For drug treatments, cells were treated with 30 μM Dyngo 4a or 1 μM Ryngo 1-23 in live cell medium for 30 min, respectively, 20 min prior to experiment. DMSO (0.001%) was used as control. For Tf uptake, cells were incubated 10 min at 37°C, 5% CO_2_ with Alexa fluor 647 conjugated Tf (5 μg/ml) followed by two washes with ice-cold PBS for a total of 15 min. Cells were immediately fixed with 3% PFA for 10 min at room temperature.

### Fluorescence microscopy analysis

The day prior to imaging, cells were induced with Dox and seeded on glass coverslips (CS-25R15) in 6-well plates at 3 × 10^5^ cells/well or on precision coverslips (No. 1.5H, Paul Marienfeld GmbH and Co. KG) in 24-well plates at 60 × 10^3^ cells/well and incubated at 37°C, 5% CO_2_. For live-cell microscopy, the media was replaced with live-cell media (DMEM high glucose, no phenol red [Gibco], supplemented with 10% FBS and 1 mM sodium pyruvate [Gibco]) and imaged at 37°C with 5% CO_2_. For TIRF-microscopy, images were acquired for 5 min at 3-s intervals with each third definitive focus using a Zeiss Axio Observer.Z1 inverted microscope that was equipped with an EMCCD camera iXon Ultra from ANDOR and an alpha Plan-Apochromat TIRF 100×/1.46 oil objective controlled by ZEN software. Confocal micrographs and stacks were acquired using a Zeiss Cell Observer Spinning Disk Confocal controlled by ZEN interface with an Axio Observer.Z1 inverted microscope, equipped with a CSU-X1A 5000 Spinning Disk Unit and an EMCCD camera iXon Ultra from ANDOR. All micrographs and acquired videos were prepared with Fiji, RRID:SCR_002285 (Schindelin et al., 2012) and Adobe Photoshop CS6, RRID:SCR_014199. For Tf-647 uptake, quantitative analysis of the Tf-647 fluorescence intensity was measured using Fiji. The fluorescent intensity was normalized to cell area instead of cell number as the cell volume was heterogenous and Dyngo-treated cells were smaller. At least 40 cells per condition were analyzed. SIM images were acquired using the Zeiss Elyra 7 with Lattice SIM^2^. The Plan-Apochromat 63×/1.4 oil DIC M27 objective was used together with a CMOS-camera PCO.edge version 4.2 CL HS controlled by ZEN software. Images were acquired every 60–120 ms as specified by respective figure legend. SIM images for Fig. 4 A were acquired using an ElyraPS1 microscope and a Plan-Apochromat 63×/1.4 oil DIC M27 objective using an Andor iXon 885 EMCCD camera with 1,004 × 1,002 pixels and 8 × 8 μm pixel size. The optimal grid size was automatically assigned to each wavelength by the Zeiss Zen software and the grid was rotated five times and shifted with five phases for each raw SIM image. Z-stacks were acquired to volumetrically image the entire structures of interest. The raw images were then processed by using the default settings for the “structured illumination processing” function in the Zen software to transform the raw images in the z-stacks into high-resolution images. Image and data precision were evaluated on 40-nm beads. A lateral precision of ∼95 and 250 nm axially was obtained. The microscope achieved lateral (X-Y) resolution of below 120 nm by resolving Gatta Quant SIM 120B DNA origami structures (Gustafsson et al., 2008).

### EM analysis

3T3-L1 fibroblasts (RRID:CVCL_0123) seeded onto glass coverslips were cultured at 37°C in a humidified CO_2_ incubator. After reaching confluence (∼3 d), the 3T3-L1 cells were induced to differentiate and cultured for 10 d as described previously (Marsh et al., 1995). PM sheets were prepared from the dorsal surface of the differentiated adipocytes by placing the glass coverslips with the cells facing down onto poly-lysine-coated EM grids (Prior et al., 2003). The grids were then detached from the glass coverslips leaving the dorsal PM of the cells (PM lawns) attached to the grids. PM lawns were then fixed with 2% PFA in PBS at room temperature and washed with PBS. Grids were immunolabeled using primary antibodies to rabbit anti-Caveolin-1 RRID:AB_397472 (610060; BD Biosciences), rabbit anti-EHD2 RRID:AB_2833022 (Morén et al., 2012), or to mouse anti-Dynamin-1/2 (MABT188; MERCK), followed by secondary labeling with goat anti-mouse gold (EM GML 10/0.25) and goat anti-rabbit gold (EM GAR10/0.25) both from BBI Solutions. Grids were contrasted using methylcellulose/uranyl acetate as described previously (Prior et al., 2003).

### Analysis of caveola dynamics

Induced HeLa FlpIn cells were imaged on TIRF over 5 min with an acquisition time of 3 s. Imaris software was used for tracking analysis of Cav1-mCh positive structures segmented as spots as previously described (Mohan et al., 2015). Slice view was used to measure the diameter of fluorescent spots in the XY plane. Structures with a diameter of 0.4 μm were tracked and the applied algorithm was based on Brownian motion with max distance traveled of 0.8 μm and a max gap size of 3. Statistics for track duration, track mean speed, track displacement length, and squared displacement were extracted and analyzed. The track duration time was not considered as absolute as a small fluctuation within the TIRF plane could produce a slight decline in fluorescent intensity over the imaging time, and thereby divide a long track into two or more shorter tracks and hence display an overrepresentation of caveolae with a short duration time. As all analyzed TIRF movies were acquired with the same microscope and on the same day within experiments, the overestimated pool of dynamic caveolae was considered as constant between samples. Tracks in figures and videos were displayed using the spectrum colormap for track duration showing the caveolae heterogeneity within a cell where red tracks represent stable caveolae and blue/purple highly dynamic caveolae (a dynamic caveolae would give rise to several tracks as it moves in and out of the TIRF plane repeated times during acquisition). Track displacement length provided information on how far the spot had moved (shortest length) from its first appearance to its last visible frame. For lateral movement analysis of the mean squared displacement, nine stable caveolae were extracted and plotted. The caveolae number per cellular basal membrane was based on the spot numbers identified by spot segmentation of the first frame from TIRF movies. At least 17 cells per condition were quantified. Colocalization of EHD2-BFP, GFP-fusion protein (EHD2, Dyn2 wild-type, or K44A) to Cav1-mCh was quantified with Imaris software. Cav1-mCh and GFP or BFP positive structures were tracked as described above for Cav1-mCh. The first three frames from a TIRF movie were analyzed, and if spots from the different channels (mCh, GFP, or BFP) were overlapping at least two out of three frames (to account for any possible discrepancy in time between the acquiring of the different channels in fast-moving caveolae), they were scored as colocalizing. A total of at least 10 cells for each condition was scored and plotted. For caveola/dynamin segmentation, each Cav1-mCh track within a 5-min TIRF movie from three cells was followed and scored for presence or no presence of Dyn2-GFP. Each track identification number was sorted into two classes; Dyn2-positive or Dyn2-negative, and the track duration time and track displacement length were extracted and plotted for each pool. For kymographic analysis, the fluorescence intensity of Dyn2-positive caveolae was extracted using Imaris. A region of interest (ROI) was made and spot analysis was performed as mentioned above. To obtain background values of the fluorescent intensity, spots were manually placed close to the area of the caveolae in both channels. After background subtraction, the mean intensity for both channels was plotted. For kymographic analysis of caveolae undergoing fission, spots were manually placed at the same position as the caveolae were last visible, and the mean intensity of 20 frames (the last 15 caveolae positive frames and 5 manually placed frames after fission) were plotted after background subtraction. Two-sample *t* test was performed on track duration (s), track displacement length (μm), and track mean speed (μm/s) data using Prism 5.0, and data are shown as fold change.

### FRAP experiments

Cells were imaged using TIRF and three reference images were recorded before an ROI containing mCh- and GFP-colocalizing structures was photobleached for 1,000 ms using maximal laser intensity (488 nm). The fluorescent recovery images were taken every 3 s for a period of 5 min. The intensities of the bleached regions were corrected for background signal and photobleaching of the cell. Data from at least 10 cells were collected per condition, and mean FRAP recovery curves were plotted using Prism 5.0 (GraphPad; RRID:SCR_002798).

### FLIM

For FLIM, HeLa FRT cells were transfected using lipofectamine, or in the case of FlpIn cell lines, induced the day before with 1 nM doxycycline to induce the expression of the protein of interest. Imaging was done on a Leica SP8 FALCON confocal, employing time-correlated single photon counting for the FLIM measurements, which enables high accuracy and high-resolution measurements. Acquisitions were done using a pulsed white light laser in the GFP excitation area at 40 Mhz as a standard and the cooled HyD1 hybrid detector. FRET on relevant pairs was calculated as a reduction of the lifetime in the green fluorophore area. Areas/spots of interest, showing overexpression, were measured using a circular ROI, and 10–20 spots were measured per cell and fitted using the LAS X software with the FLIM plugin. Data were plotted using Prism 5.0. FLIM and FRET images were pixel-wise fitted using the LAS X software and exported to tif. Final adjustments were done using Fiji, where images were normalized to each other to show the increase/decrease of lifetime and FRET between the images.

### Immunostaining

Induced Dyn2-GFP-Cav1-mCh HeLa cells were transfected with the EHD2-BFP expression vector and seeded on precision coverslips (No. 1.5H, Paul Marienfeld GmbH and Co. KG) in 24-well plates at 50 × 10^3^ cells/well and incubated overnight (37°C, 5% CO_2_). Following incubation with Dyngo 4a (30 μM) or DMSO (0.001%) for 30 min, cells were fixed with 3% PFA in PBS (Electron Microscopy Sciences) and subsequent permeabilization and blocking was carried out simultaneously using PBS containing 5% goat serum and 0.05% saponin. Cells were then immunostained with rabbit anti-PTRF, RRID:AB_88224 (Abcam) followed by goat anti-rabbit IgG secondary antibody coupled to Alexa Fluor 647, RRID:AB_2535814 (Thermo Fisher Scientific) as previously described (Lundmark et al., 2008). Confocal images were acquired using the Zeiss Spinning Disk Confocal microscope (63× lens). Micrographs were prepared using Fiji (Schindelin et al., 2012) and Adobe Photoshop CS6.

### Statistical analysis

Two-sample *t* test was used to compare values in the different experiments using the GraphPad prism program version 5.0. P < 0.05 was considered a statistically significant change. *P < 0.05; **P < 0.01; ***P < 0.001; NS, not significant. All the values were presented as mean ± SEM as specified in the figure legends.

### Online supplementary material

Fig. S1 shows mean squared displacement of stable caveolae and the uptake of Tf-647 in Cav1-mCh HeLa FlpIn cells treated with ctrl or Dyn2 siRNA as well as caveolae dynamic parameters following Dyn1 depletion. Fig. S2 provides additional data on the colocalization of Dyn2-GFP and Cav1-mCh imaged by SIM. Fig. S3 shows Dyn2-GFP localization after EHD2 or Pac2 depletion and characterization of GFP-EHD2-Cav1-mCh and Pac2-GFP-Cav1-mCh HeLa FlpIn cells as well as representative FLIM-FRET images of GFP-fusion proteins and Cav1-mCh in FlpIn HeLa cells. Fig. S4 shows the protein titration of the HeLa FlpIn cell lines expressing K44A Dyn2-GFP and Cav1-mCh or I684K Dyn2-GFP and Cav1-mCh after Dox induction. Fig. S5 provides additional information on the effect that Dyngo 4a has on the Dyn2-GFP-Cav1-mCh HeLa FlpIn cells. Video 1 shows single particle tracking of Cav1-mCh in a Cav1-mCh HeLa FlpIn cell treated with ctrl siRNA imaged on TIRF. Video 2 shows single particle tracking of Cav1-mCh in a Cav1-mCh HeLa FlpIn cell treated with EHD2 siRNA imaged on TIRF. Video 3 shows single particle tracking of Cav1-mCh in a Cav1-mCh HeLa FlpIn cell treated with Dyn2 siRNA imaged on TIRF. Video 4 shows live cell imaging of a Dyn2-GFP-Cav1-mCh HeLa FlpIn cell using SIM (SIM^2^ algorithm). Video 5 shows live-cell TIRF imaging of Dyn2-GFP-Cav1-mCh HeLa FlpIn cells treated 30 min with 30 μΜ οf Dyngo 4a.

## Supplementary Material

SourceData F1 contains original blots for Fig. 1.

SourceData F2 contains original blots for Fig. 2.

SourceData FS1 contains original blots for Fig. S1.

SourceData FS3 contains original blots for Fig. S3.

SourceData FS4 contains original blots for Fig. S4.
