# Supplementary figures and images for: Dynamin2 functions as an accessory protein to reduce the rate of caveola internalization
Source: J Cell Biol. 2023 Feb 2;222(4):e202205122. doi: 10.1083/jcb.202205122 (PMC9929934; doi:10.1083/jcb.202205122)

Blots for Figure1 C

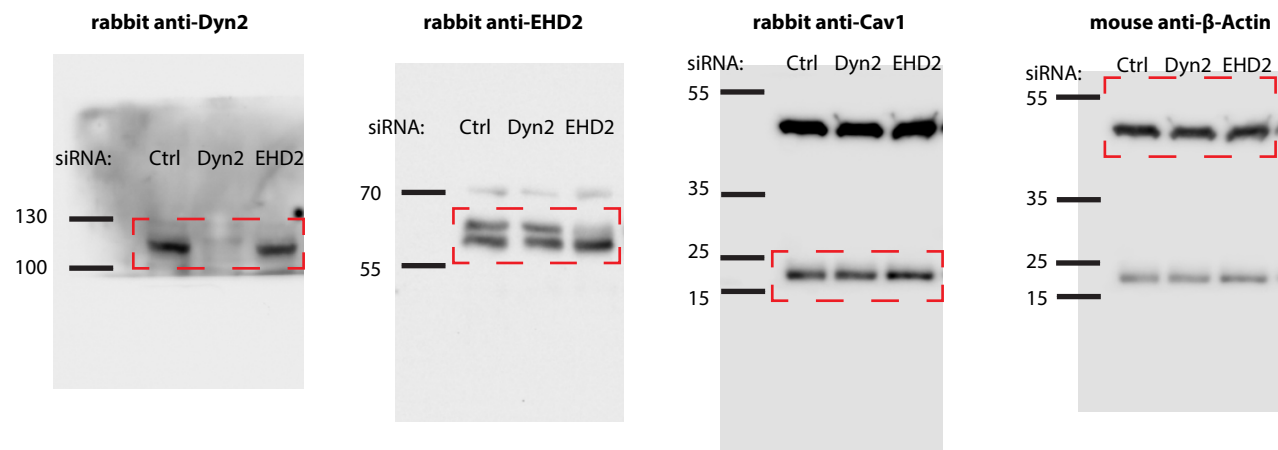

Supplement: SourceData F1 — contains original blots for Fig. 1. [file JCB_202205122_SourceDataF1.pdf]

Blots for Figure 2A

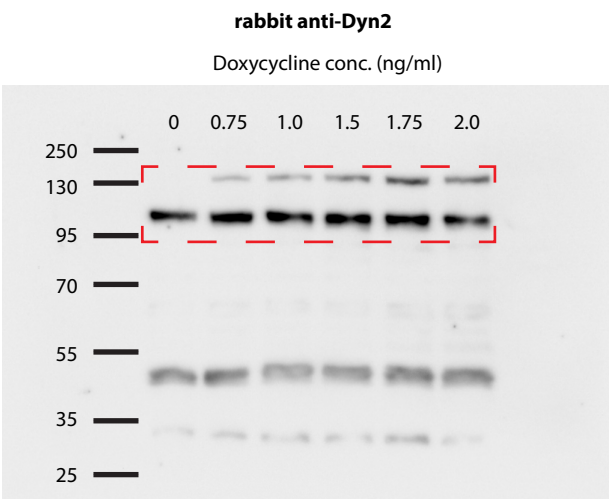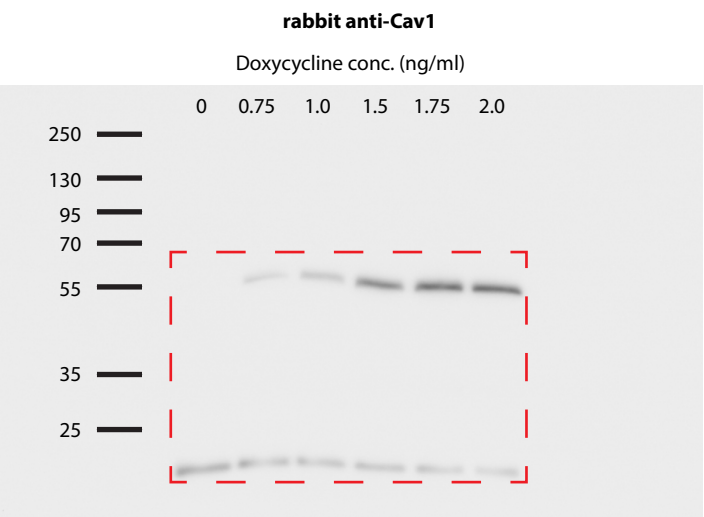

Supplement: SourceData F2 — contains original blots for Fig. 2. [file JCB_202205122_SourceDataF2.pdf]

Blots for Figure S1D

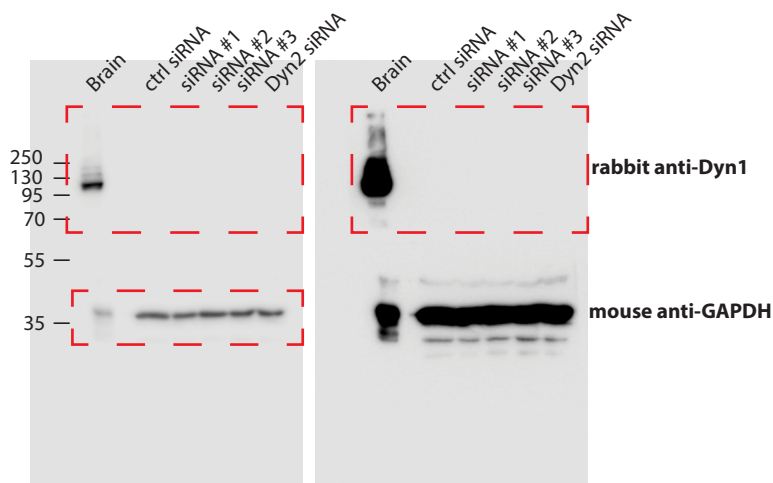

Supplement: SourceData FS1 — contains original blots for Fig. S1. [file JCB_202205122_SourceDataFS1.pdf]

Blots for Figure S3

A

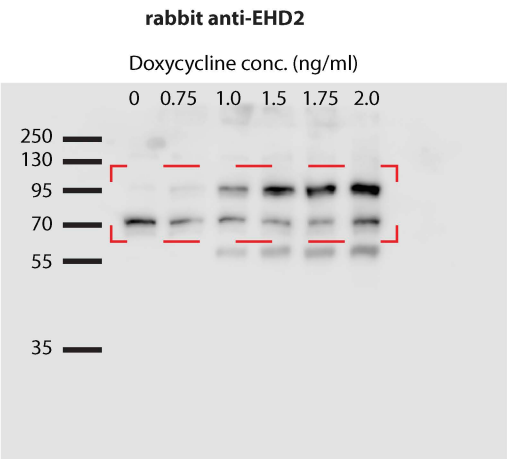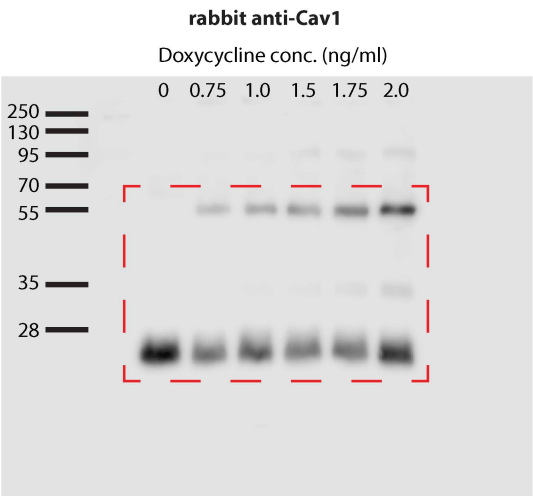

B

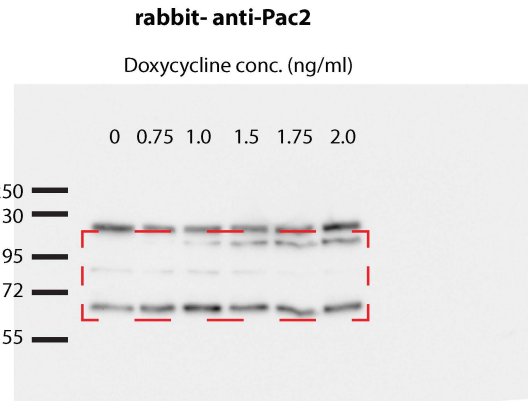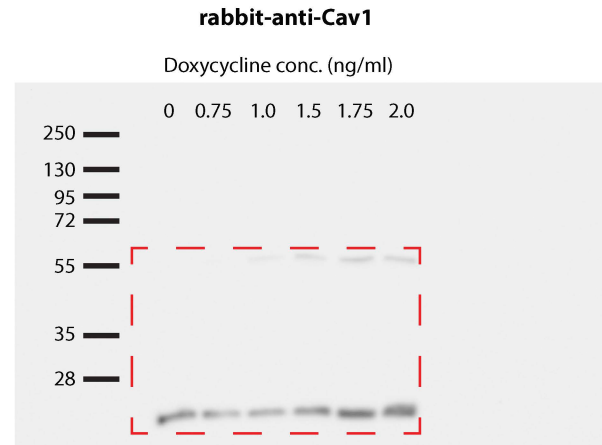

C

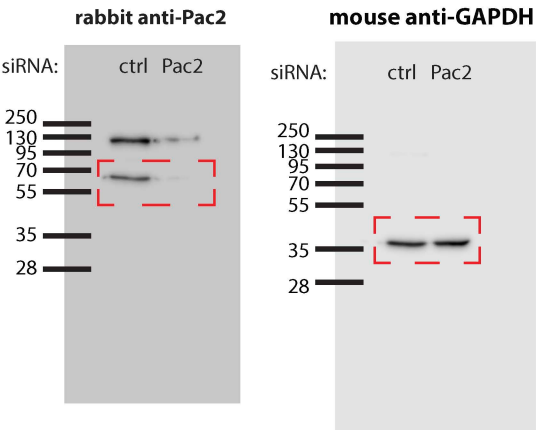

Supplement: SourceData FS3 — contains original blots for Fig. S3. [file JCB_202205122_SourceDataFS3.pdf]

**Blots for Figure S4**

**B**

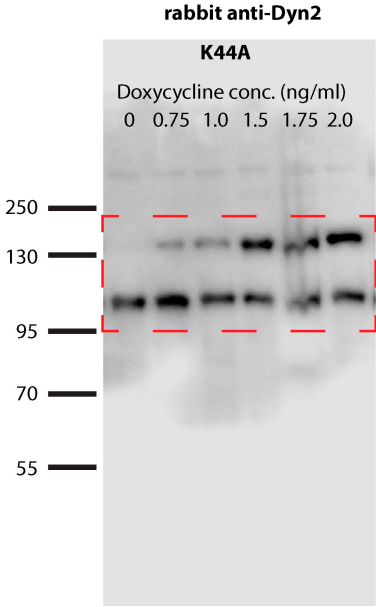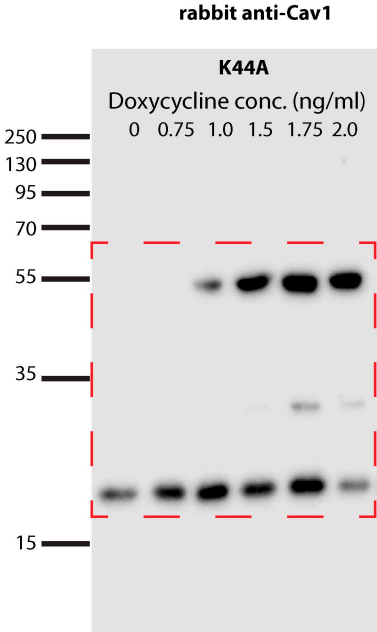

**D**

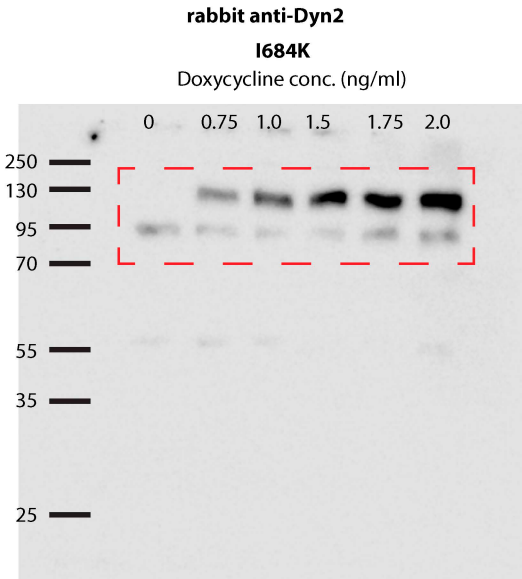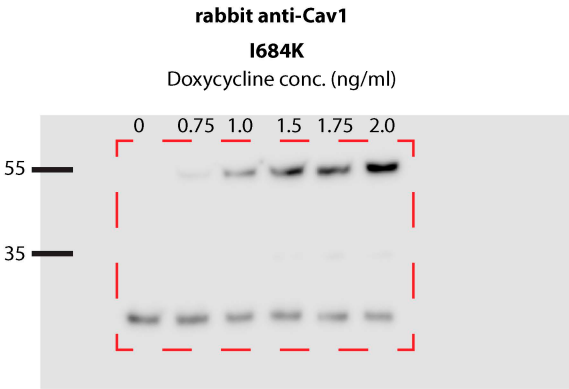

Supplement: SourceData FS4 — contains original blots for Fig. S4. [file JCB_202205122_SourceDataFS4.pdf]
